# Parasite in cancer therapy: molecular mechanisms and translational potential

**DOI:** 10.3389/fimmu.2026.1901126

**Published:** 2026-07-17

**Authors:** Juanyan Liao, Xingyu Hou, Haolin Tang, Qing Li

**Affiliations:** 1 Department of Biotherapy, Cancer Center, West China Hospital, Sichuan University, Chengdu, Sichuan, China; 2 West China School of Medicine, West China Hospital, Sichuan University, Chengdu, Sichuan, China

**Keywords:** antitumor mechanisms, cancer therapy, immunomodulation, parasite-derived molecules, translational potential, tumor microenvironment

## Abstract

Parasite-derived molecules have emerged as a promising source of natural bioactive compounds with immunomodulatory and antitumor properties, attracting increasing attention in cancer research. Derived from both protozoan and helminth parasites, these molecules exhibit diverse biological activities that extend beyond parasite survival and represent a novel resource for cancer therapy. Accumulating evidence demonstrates that parasite-derived molecules suppress tumor progression through complementary immune-mediated and non-immune mechanisms, including activation of innate and adaptive antitumor immunity, remodeling of the tumor microenvironment, induction of apoptosis and autophagy, inhibition of angiogenesis and metastasis, and regulation of tumor metabolism. Recent preclinical studies have demonstrated encouraging therapeutic efficacy across multiple tumor models, including melanoma, lung cancer, colorectal cancer, breast cancer, hepatocellular carcinoma, and other malignancies. In addition to summarizing the major classes of parasite-derived molecules and their mechanisms of action, this review highlights recent advances in translational research, including combination therapeutic strategies, immunogenicity and safety, delivery system optimization, and manufacturing and regulatory considerations. Despite encouraging preclinical findings, substantial challenges remain before clinical translation can be achieved. By integrating current mechanistic evidence with emerging translational perspectives, this review provides a comprehensive overview of parasite-derived molecules as potential anticancer agents and offers insights to facilitate their future development and clinical application in cancer therapy.

## 1 Introduction

Despite substantial advances in cancer diagnosis and treatment, cancer remains a major global health challenge worldwide. Cancer immunotherapy has revolutionized the treatment landscape for multiple malignancies over the past decade. Although surgery, chemotherapy, radiotherapy, targeted therapy, and immunotherapy have significantly improved clinical outcomes in many malignancies, durable therapeutic responses remain limited in a considerable proportion of patients because of tumor heterogeneity, immune evasion, and the immunosuppressive tumor microenvironment (TME) (1). Consequently, the identification of novel immunomodulatory agents capable of enhancing antitumor immunity while minimizing systemic toxicity has become an important direction in cancer research (2).

In recent years, parasite-derived molecules (PDMs) have attracted increasing attention as a unique source of bioactive compounds with diverse immunoregulatory and antitumor properties (2–4). Owing to the long-term co-evolution between parasites and their hosts, these molecules have evolved sophisticated strategies to modulate host immune responses and cellular signaling pathways, providing valuable resources for the development of innovative cancer therapeutics (4–6).

Parasites have evolved intricate host-adaptation strategies through long-term co-evolution with their hosts, enabling them to establish persistent infections while maintaining a finely balanced host–parasite relationship (4–6). Central to this process is the secretion or expression of a broad spectrum of bioactive molecules, including proteins, glycoconjugates, peptides, nucleic acids, and extracellular vesicles, which regulate innate and adaptive immune responses and influence diverse cellular signaling pathways (5, 7, 8). Increasing evidence indicates that many of these molecules possess biological activities extending beyond parasite survival, including modulation of immune cell function, regulation of inflammatory responses (5, 8), inhibition of angiogenesis (9), induction of tumor cell apoptosis (10–12), and remodeling of the TME (13, 14), thereby providing a mechanistic basis for their potential application in cancer therapy (2, 3).

Importantly, PDMs originate from two major parasite groups—protozoa and helminths—which differ substantially in their biological characteristics, secretomes, and host-interaction strategies (4–6). These differences contribute to distinct immunomodulatory properties and antitumor activities, underscoring the importance of systematic classification when evaluating their therapeutic potential (3, 5).

Accumulating evidence from experimental studies has demonstrated that PDMs exert antitumor effects through both immune-mediated and non-immune mechanisms (2–4). On the one hand, these molecules can activate dendritic cells (DCs), macrophages, natural killer cells, and cytotoxic T lymphocytes (2, 13, 14), while modulating cytokine production and immune checkpoint signaling to enhance antitumor immunity and reverse the immunosuppressive TME (2, 13, 14). On the other hand, PDMs directly influence tumor cell biology by inducing apoptosis and autophagy (10, 11, 15–17), suppressing angiogenesis (9, 18), inhibiting invasion and metastasis (9, 12), and reprogramming tumor metabolism (16), thereby limiting tumor progression through multiple complementary pathways. These multifaceted biological activities highlight the potential advantages of PDMs over conventional single-target therapeutic strategies and provide a strong mechanistic foundation for their further translational development in oncology (2, 13).

The rapid expansion of preclinical research has further highlighted the broad applicability of PDMs across multiple cancer types. Experimental studies have demonstrated encouraging antitumor activity in melanoma (19), lung cancer (10), colorectal cancer (CRC) (16, 17), breast cancer (20), hepatocellular carcinoma (18), several other solid tumors (21, 22), as well as hematological malignancies (23). These investigations collectively indicate that PDMs can inhibit tumor growth, suppress metastatic dissemination, improve antitumor immune responses, and enhance the therapeutic efficacy of conventional treatments under diverse experimental settings. Although most evidence remains at the preclinical stage, the increasing diversity of PDMs and tumor models investigated has substantially expanded current understanding of their therapeutic potential and provided a rationale for future clinical translation (2, 13, 14). In summary, these studies indicate that PDMs exhibit broad-spectrum antitumor activity across diverse tumor models, although further validation in clinical settings remains necessary.

Despite these encouraging advances, several challenges continue to impede the clinical translation of PDMs. Issues including immunogenicity, biosafety, large-scale production, quality control, delivery efficiency, and regulatory standardization must be systematically addressed before these molecules can be successfully integrated into clinical practice (2, 13). In addition, the considerable biological diversity of PDMs and the complexity of tumor heterogeneity underscore the need for further mechanistic investigations, optimized delivery strategies, and well-designed translational studies (3, 14). In this review, we systematically summarize the major classes of PDMs from protozoa and helminths, discuss their immune-mediated and non-immune antitumor mechanisms, highlight recent advances across different cancer types, and examine current opportunities and challenges for their clinical translation. By integrating mechanistic insights with translational perspectives, this review aims to provide a comprehensive overview of current evidence and facilitate the future development and clinical translation of PDM-based cancer therapeutics. For clarity, PDMs discussed in this review are broadly classified into protozoan-derived molecules and helminth-derived molecules according to standard parasitological taxonomy.

## 2 Literature search strategy

This review was conducted as a narrative literature review to summarize current advances in the antitumor effects of PDMs and their translational potential in cancer therapy. Relevant literature published up to June 2026 was retrieved from PubMed, Web of Science Core Collection, and Scopus, with additional studies identified through manual screening of the reference lists of relevant publications.

The search strategy combined Medical Subject Headings (MeSH) and free-text terms, including “parasite-derived molecules,” “parasite-derived proteins,” “Toxoplasma gondii,” “Plasmodium,” “Leishmania,” “Schistosoma,” “Trichinella,” “Echinococcus,” “cancer,” “tumor microenvironment,” “immunotherapy,” “apoptosis,” “autophagy,” “angiogenesis,” “metastasis,” and “metabolism”.

This review primarily summarizes experimentally validated studies investigating the antitumor activities of PDMs, including mechanistic studies, preclinical investigations, and available clinical evidence, with original research articles prioritized whenever possible. PDMs were classified according to standard parasitological taxonomy into protozoan- and helminth-derived molecules. The review focuses on parasite species for which substantial experimental evidence supports antitumor activity, whereas parasites with only limited or indirect evidence were not discussed in detail. The overall organization of this review follows the sources of PDMs, their mechanisms of action, evidence across different cancer types, and their translational potential.

## 3 Types and sources of parasite-derived molecules

PDMs discussed in this review originate from two major taxonomic groups: protozoa and helminths. Despite their distinct biological characteristics, both groups produce diverse bioactive molecules capable of modulating antitumor immunity and tumor-associated biological processes (3–5). Representative molecules are summarized below according to this taxonomic classification (2).

### 3.1 Protozoan-derived molecules

#### 3.1.1 Toxoplasma gondii

*Toxoplasma gondii* produces a diverse repertoire of bioactive molecules, among which dense granule proteins (GRAs) and rhoptry proteins (ROPs) are the most extensively investigated for their immunomodulatory and antitumor activities. Representative molecules, including GRA7, GRA12, GRA15, and ROP18, have demonstrated potent immunostimulatory properties by activating innate immune cells and promoting Th1-polarized immune responses characterized by increased production of interleukin-12 (IL-12) and interferon-γ (IFN-γ) both of which are essential for effective antitumor immunity (24–26).

Among these molecules, recombinant GRA12 encapsulated in poly lactic-co-glycolic acid nanoparticles induced robust Th1-biased immune responses, enhanced lymphocyte proliferation, and elevated IFN-γ secretion in experimental models (24), whereas GRA7 promoted DC and macrophage activation, thereby facilitating antigen presentation and pro-inflammatory cytokine production (24). GRA15 further enhanced IL-12 production through activation of macrophages (26), while ROP18 regulated host inflammatory signaling through the IL20RB/JAK–STAT3 pathway, highlighting the diverse immunomodulatory functions of *Toxoplasma gondii*–derived molecules (25). Although ROP18 has also been implicated in immune evasion and tumor-promoting effects under chronic infection, its biological activity appears to depend on the infection status and molecular context (27).

Accumulating evidence from melanoma, breast cancer, and CRC models has demonstrated that *Toxoplasma gondii*–derived extracts or recombinant proteins suppress tumor growth through multiple mechanisms (17, 20, 28, 29). These include activation of DC and macrophages (13, 20), polarization of macrophages toward the M1 phenotype (14), induction of tumor cell apoptosis (17, 28), inhibition of angiogenesis (28), and reversal of tumor-induced immunosuppression (14, 29). Taken together, these findings identify GRA and ROP proteins as representative *Toxoplasma gondii*–derived bioactive molecules with considerable potential for cancer immunotherapy, while emphasizing the need to optimize their safety and efficacy through recombinant proteins or attenuated non-replicating platforms before clinical translation (2, 4) (**Table 1**).

**Table 1:** Representative parasite-derived molecules and their antitumor properties.

Parasite	Representative molecules	Major antitumor mechanisms	Representative cancer models	Key signaling pathways	References
Protozoan parasites					
*Toxoplasma gondii*	GRA7, GRA12, GRA15, ROP18	Th1 immune activation; dendritic cell and macrophage activation; M1 macrophage polarization; apoptosis; anti-angiogenesis; reversal of tumor immunosuppression	Melanoma, breast cancer, colorectal cancer	IL20RB/JAK–STAT3	(2, 4, 13, 14, 17, 20, 24–26, 28, 29)
*Plasmodium* spp.	GPI, hemozoin, parasite nucleic acids, immunogenic parasite proteins	DC, NK and CD8^+^ T-cell activation; immune microenvironment remodeling; inhibition of tumor angiogenesis; macrophage polarization; hypoxia-associated signaling regulation	Lung cancer, hepatocellular carcinoma	PRR/TLR, HIF-1α	(4, 14, 18, 30–32)
*Leishmania* spp.	LPG, GP63, extracellular vesicles	Immune modulation; macrophage/DC regulation; chemokine signaling modulation; apoptosis; autophagy; tumor immune microenvironment regulation	Liver cancer, breast cancer	TLR2/4, NOD2	(4, 14, 15, 33–42)
Helminth parasites					
*Schistosoma* spp.	SEA, SWAP, IPSE/α-1, Omega-1	Th2/Treg modulation; immune microenvironment remodeling; apoptosis; anti-angiogenesis; reduced tumor cell migration; chemosensitization	Colorectal cancer	Apoptosis-related signaling; angiogenesis-related pathways	(8, 9, 14, 43–47)
*Trichinella spiralis*	ES antigens	Balanced Th1/Th2 responses; cytotoxic T-cell activation; apoptosis; tumor immune microenvironment modulation	Lung cancer	Immune regulation	(10, 11, 48)
*Fasciola hepatica*	ES antigens; Cathepsin L	Reduced tumor cell viability; apoptosis; cell-cycle arrest; extracellular matrix remodeling (potential)	Lung cancer (A549)	ROS-related signaling; ECM remodeling	(2, 49–51)
*Ascaris lumbricoides*	ES antigens; Al-CPI	Immunomodulation; macrophage activation; anti-inflammatory activity; inflammatory cytokine regulation; reduced colorectal cancer cell viability	Colorectal cancer	Macrophage activation; inflammatory cytokine regulation	(46, 52–54)
*Echinococcus granulosus*	EgKI-1; Kunitz4-derived peptides; ES products; HCF	NK-cell activation; Th1 polarization; apoptosis; EAG1 blockade; immunological cross-reactivity; context-dependent pro-tumor effect	Lung cancer	EAG1, p27, p53	(55–58)

#### 3.1.2 *Plasmodium* spp.

Molecules derived from *Plasmodium* spp. have attracted increasing interest because of their potent immunostimulatory properties and potential applications in cancer immunotherapy (4, 14). Representative *Plasmodium*-derived molecules and pathogen-associated molecular patterns, including glycosylphosphatidylinositol (GPI), hemozoin, parasite nucleic acids, and immunogenic parasite proteins, activate innate immune signaling primarily through pattern-recognition receptors (PRRs), particularly Toll-like receptors (TLRs), thereby providing an immunological basis for their potential antitumor applications (4, 30, 31).

Experimental studies have demonstrated that *Plasmodium* infection or parasite-derived components activate DCs, natural killer (NK) cells, and cytotoxic CD8+ T cells, accompanied by increased production of IFN-γ and other pro-inflammatory cytokines (18, 30, 32). These immune responses remodel the tumor immune microenvironment, enhance immune surveillance, and overcome tumor-induced immunosuppression, ultimately suppressing tumor progression (14, 18, 32). In murine models of lung cancer and hepatocellular carcinoma, *Plasmodium* infection and parasite-derived components significantly inhibited tumor growth and prolonged survival through both direct antitumor effects and activation of host antitumor immunity (18, 32).

In addition to immune activation, *Plasmodium*-derived molecules have also been reported to regulate tumor-associated signaling pathways involved in macrophage polarization, angiogenesis, and hypoxia, thereby contributing to their antitumor activities (18, 32). Overall, these findings identify *Plasmodium*-derived molecules as promising immunomodulatory agents for cancer therapy (4, 14). Further characterization of their bioactive components and optimization of delivery strategies will facilitate their future translational development (2, 13) (**Table 1**).

#### 3.1.3 *Leishmania* spp.

Molecules derived from *Leishmania* spp. possess diverse immunomodulatory activities that contribute to their potential applications in cancer therapy (4, 33). Among them, lipophosphoglycan (LPG) and glycoprotein 63 (GP63) are the best-characterized bioactive molecules (4, 34). LPG, a major surface glycoconjugate, regulates host immune responses through interactions with TLRs (35), whereas GP63, a zinc-dependent metalloprotease, facilitates host–parasite interactions (36).

LPG and GP63 exhibit multifaceted immunoregulatory functions by influencing DCs, macrophages, and γδ T cells (4, 33), thereby regulating cytokine production through TLR2-, TLR4-, and NOD2-dependent pathways (35). In addition, GP63 contributes to immune modulation by interfering with chemokine signaling (37), while extracellular vesicles containing LPG and GP63 further regulate macrophage polarization (38, 39) and host immune responses (40, 41). These immunomodulatory properties provide a mechanistic basis for exploiting *Leishmania*-derived molecules as therapeutic agents for cancer (4, 15).

Experimental studies have demonstrated that *Leishmania*-derived molecules suppress tumor growth in liver cancer and breast cancer models by inducing tumor cell apoptosis and autophagy while modulating the tumor immune microenvironment (15, 42). However, the immunosuppressive properties of certain *Leishmania* molecules may also limit their therapeutic application in specific tumor settings, emphasizing the importance of optimizing their biological activity and delivery strategies before clinical translation (4, 14) (**Table 1**).

### 3.2 Helminth-derived molecules

**Figure 1:**
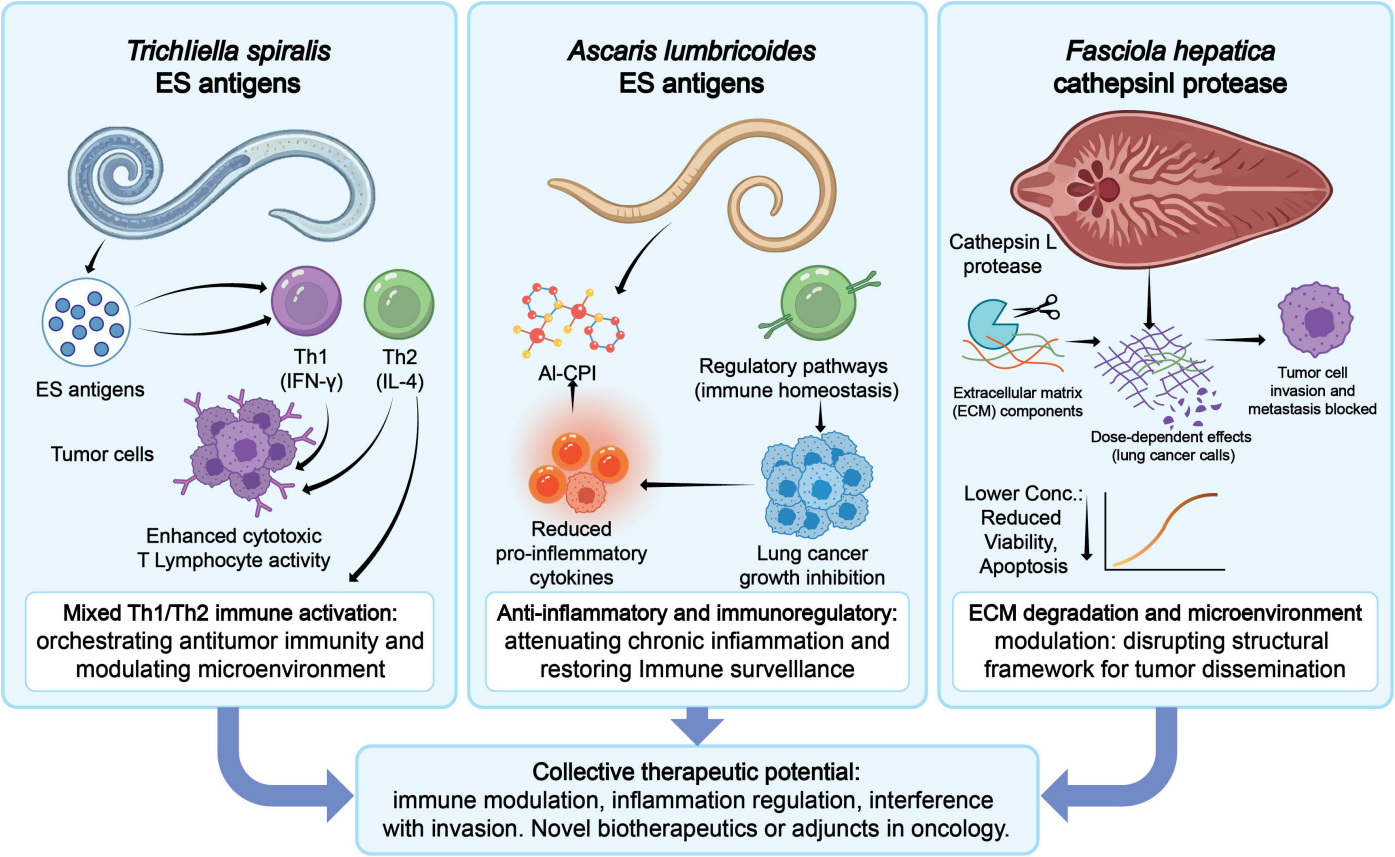
Representative antitumor mechanisms of selected helminth-derived molecules. *Trichinella spiralis* excretory-secretory (ES) antigens induce balanced Th1/Th2 responses, enhancing cytotoxic T-cell activity and suppressing tumor growth in lung cancer models. *Ascaris lumbricoides* ES antigens reduce colorectal cancer cell viability and exhibit immunomodulatory properties. *Fasciola* hepatica ES antigens reduce tumor cell viability and induce apoptosis, while Cathepsin L is a major component of parasite ES products with extracellular matrix-remodeling activity.

Helminths produce diverse bioactive molecules with immunomodulatory and antitumor properties (5, 6, 8), and representative antitumor mechanisms of selected helminth-derived molecules are summarized in **Figure 1**.

#### 3.2.1 *Schistosoma* spp.

Molecules derived from *Schistosoma* spp., particularly soluble egg antigens (SEA) and soluble worm antigen preparations (SWAP), possess diverse immunomodulatory activities with potential applications in cancer therapy (8, 14, 43). Representative bioactive molecules, including IPSE/α-1 and Omega-1, regulate host immune responses by promoting Th2 polarization (8) and modulating regulatory T-cell (Treg) functions (44), thereby reshaping the tumor immune microenvironment (14).

Beyond their immunoregulatory functions, schistosome-derived molecules exhibit direct antitumor activities in experimental models of cancers (45–47). *Schistosoma*-derived molecules suppress tumor growth by inhibiting angiogenesis and reducing tumor cell migration (9) while inducing apoptosis through multiple molecular mechanisms (45–47). These molecules also modulate the tumor immune microenvironment by regulating inflammatory responses (8) and enhancing immune-mediated tumor suppression (14), highlighting their multifaceted antitumor mechanisms.

In addition, accumulating evidence suggests that schistosome-derived molecules appear to enhance tumor cell sensitivity to conventional chemotherapy by regulating pathways associated with apoptosis, cell survival, and drug resistance (47). The combination of their immunomodulatory (8, 43), antiangiogenic (9), and chemosensitizing properties (47) suggests considerable translational potential for *Schistosoma*-derived molecules as adjuncts to current cancer therapies (14, 45). Nevertheless, the precise molecular targets and mechanisms underlying these effects remain to be fully elucidated (**Table 1**).

#### 3.2.2 Trichinella spiralis

Molecules derived from *Trichinella spiralis*, particularly excretory-secretory (ES) antigens, exhibit potent immunomodulatory properties with potential applications in cancer therapy (10, 11). ES antigens induce a balanced Th1/Th2 immune response, characterized by enhanced production of IFN-γ and IL-4, thereby coordinating both cellular and humoral immunity (11).

Experimental studies have demonstrated that *Trichinella spiralis* ES antigens suppress tumor growth in lung cancer models by enhancing cytotoxic T-cell activity, modulating the tumor immune microenvironment, and promoting tumor cell apoptosis (10, 11, 48). The coordinated activation of Th1- and Th2-associated immune responses is considered to contribute to effective antitumor immunity while maintaining immune homeostasis (11). Although the precise bioactive components and molecular mechanisms remain to be fully characterized, *Trichinella spiralis*-derived ES antigens represent promising immunomodulatory candidates for future cancer immunotherapy (**Table 1**).

#### 3.2.3 Fasciola hepatica

Molecules derived from *Fasciola hepatica*, particularly the cysteine protease cathepsin L (CatL), exhibit promising antitumor activities through modulation of the TME (2, 49). CatL is a major component of parasite ES products and possesses potent proteolytic activity capable of remodeling the extracellular matrix (ECM), thereby influencing tumor invasion and metastasis (50, 51).

Experimental studies have demonstrated that *Fasciola hepatica* ES antigens suppress tumor cell viability, induce apoptosis, and interfere with cell-cycle progression in a dose-dependent manner (49). In addition, CatL-mediated modulation of ECM dynamics has been shown to impair tumor cell migration and metastatic dissemination, highlighting its potential role in limiting tumor progression (51). Although the extracellular matrix-remodeling activity of CatL suggests a potential influence on tumor cell migration and metastatic dissemination, direct evidence supporting this mechanism in cancer remains limited (51). Further studies are therefore required to elucidate its molecular targets and therapeutic potential. Nevertheless, *Fasciola hepatica*-derived molecules represent promising candidates for the development of novel anticancer therapeutics (**Table 1**).

#### 3.2.4 Ascaris lumbricoides

Molecules derived from *Ascaris lumbricoides*, including ES antigens and immunomodulatory proteins such as Al-CPI, exhibit anti-inflammatory and immunomodulatory properties with potential applications in cancer therapy (52–54). Al-CPI regulates host immune responses by modulating macrophage activation and inflammatory signaling, thereby promoting immune homeostasis (53, 54). Preliminary studies have demonstrated that *Ascaris lumbricoides* ES antigens reduce CRC cell viability *in vitro*, suggesting direct antitumor activity (52). In addition, parasite-associated immune modulation may influence the tumor immune microenvironment through regulation of inflammatory cytokines and host immune responses, providing a potential mechanistic basis for future cancer immunotherapy (46, 53, 54). Although the active components and molecular mechanisms remain to be fully elucidated, *Ascaris lumbricoides*–derived molecules represent promising candidates for further investigation as immunomodulatory agents in cancer therapy (52–54) (**Table 1**).

#### 3.2.5 *Echinococcus* spp.

Molecules derived from *Echinococcus* spp., particularly *Echinococcus granulosus*, have recently emerged as promising candidates for cancer therapy owing to their immunomodulatory and antitumor activities (55, 56). Among these, Kunitz-type protease inhibitor EgKI-1 and its derived peptides are the best-characterized bioactive molecules (57). Recent studies have demonstrated that Kunitz4-derived peptides inhibit cancer cell proliferation and induce apoptosis by blocking the EAG1 potassium channel, leading to G0/G1 cell-cycle arrest and modulation of p27- and p53-associated signaling pathways (57).

In addition to purified bioactive molecules, ES products and hydatid cyst fluid (HCF) have also been shown to enhance antitumor immunity by activating NK cells, promoting Th1-polarized immune responses, and eliciting immunological cross-reactivity with tumor-associated antigens, thereby suppressing tumor growth in experimental models (55, 56). However, recent evidence suggests that certain HCF components may promote lung cancer cell proliferation under specific experimental conditions, indicating that the biological activities of *Echinococcus*-derived molecules are context-dependent (58). Overall, *Echinococcus*-derived molecules represent promising candidates for future cancer immunotherapy, although further studies are required to identify their key bioactive components and clarify their mechanisms before clinical translation (**Table 1**).

## 4 Immune-mediated antitumor mechanisms

### 4.1 Innate immune response activation

PDMs initiate antitumor immunity primarily by activating innate immune responses, which constitute the first line of host defense against both pathogens and malignant cells (2, 3). These molecules interact with PRRs, including TLRs and nucleotide-binding oligomerization domain-containing proteins (NODs), expressed on antigen-presenting cells (APCs) such as DCs, macrophages, and NK cells (7, 35, 59). Activation of TLR2, TLR4, and NOD2 triggers downstream signaling cascades that promote DC maturation, macrophage activation, NK-cell cytotoxicity, and the production of pro-inflammatory cytokines, including IL-12, IFN-γ, and tumor necrosis factor-α (TNF-α) (7, 35, 59). This inflammatory milieu facilitates the recruitment and activation of effector immune cells, thereby establishing an immune microenvironment favorable for tumor elimination (2, 7).

For example, filarial PDMs regulate DC function through TLR signaling, subsequently shaping CD4^+^ T-cell responses and maintaining immune balance between pro- and anti-inflammatory states, illustrating the potent immunomodulatory capacity of PDMs (7). In summary, these early innate immune events provide a critical immunological foundation for subsequent adaptive antitumor responses (2, 3).

In addition to membrane-associated PRRs, PDMs also activate cytosolic innate immune sensing pathways. A representative example is provided by *Toxoplasma gondii* dense granule antigens (GRA proteins), which have been associated with activation of type I interferon responses through innate immune sensing pathways, thereby enhancing host antiviral-like and antitumor immune programs (2, 25). Type I interferons enhance antigen presentation by APCs while promoting the cytotoxic activity of NK cells and CD8^+^ T cells, thereby strengthening both innate and adaptive antitumor immunity (2, 3).

Likewise, nucleic acids derived from *Plasmodium* spp. activate plasmacytoid dendritic cells (pDCs) primarily through TLR9-mediated signaling pathways (4, 60). Recognition of parasite-derived CpG DNA motifs induces type I interferon production and additional pro-inflammatory cytokines, thereby enhancing immune surveillance against tumor cells while contributing to parasite clearance (4, 60).

Beyond direct receptor activation, parasite-derived extracellular vesicles (EVs) and secreted proteins further modulate innate immune cell function. EVs released from *Leishmania* spp. carry diverse PDMs capable of reshaping host immune responses (38, 39, 41). These vesicles can influence macrophage polarization and inflammatory signaling, highlighting their role in tumor-associated immune remodeling (14, 39).

PDMs also regulate other innate immune populations. Mast cells (MCs), which participate in host defense through degranulation, cytokine secretion, and inflammatory amplification, can likewise be modulated by PDMs (61). Although their contribution to parasite-mediated antitumor immunity remains incompletely understood, their immunoregulatory properties warrant further investigation as potential targets for cancer immunotherapy (2, 61).

PDMs additionally exert systemic immunomodulatory effects that may indirectly support antitumor immunity. Helminth-derived molecules, including those from *Fasciola hepatica*, have been reported to modulate phosphoinositide 3-kinase/protein kinase B (PI3K/Akt) signaling pathways and inflammatory responses, although these effects have primarily been investigated in non-cancer disease models (62, 63). This highlights the broader capacity of helminth-derived molecules to regulate immune homeostasis, suggesting potential relevance for cancer-associated inflammation (63, 64).

Similarly, helminth-derived excretory-secretory products suppress systemic inflammation, improve intestinal barrier function, and reshape gut microbiota composition (63, 65, 66). Taken together, these findings demonstrate that PDMs activate innate immunity through multiple complementary mechanisms, including PRR signaling, cytosolic immune sensing, immune-cell reprogramming, and systemic immunomodulation, thereby providing a strong mechanistic basis for their further development as cancer immunotherapeutic agents (2, 4, 13).

### 4.2 Adaptive immune responses activation

PDMs promote antitumor immunity not only through innate immune activation but also by contributing to adaptive immune responses (3, 7, 67). A central mechanism involves the regulation of APCs, particularly DCs, which may enhance tumor antigen processing and presentation, thereby facilitating subsequent activation of tumor-specific CD8^+^ T-cell responses (7, 59, 67). Upon stimulation by PDMs, DCs upregulate major histocompatibility complex (MHC) molecules and co-stimulatory molecules, including CD80 and CD86, while producing cytokines such as IL-12, which collectively support efficient T-cell priming and expansion (7, 35, 59, 67). These coordinated events contribute to the development of cytotoxic T-lymphocyte responses that are associated with tumor cell elimination (2, 4).

Among helminth-derived molecules, omega-1, a major glycoprotein presents in the SEA of *Schistosoma* spp., represents a well-characterized immunomodulatory factor (43, 68). Although omega-1 is classically associated with Th2-polarized immune responses characterized by IL-4, IL-5, and IL-13 production, accumulating evidence suggests that its effects on DC function may influence downstream T-cell priming within different immunological contexts (68, 69). In the TME, such modulation may contribute to shaping antigen presentation and influencing adaptive immune responses, thereby potentially affecting antitumor immunity (2, 3, 67).

Surface LPG from *Leishmania* spp. provides another representative example of parasite-mediated adaptive immune regulation. LPG interacts with pattern-recognition receptors, particularly TLR2, on DCs, leading to activation of intracellular signaling pathways such as mitogen-activated protein kinase (MAPK) and NF-κB, which support DC maturation, cytokine production, and upregulation of co-stimulatory molecules (35, 59, 70). These changes are associated with the polarization of CD4^+^ T cells toward a Th1-type immune response characterized by IFN-γ production, which contributes to enhanced macrophage activation and may support CD8^+^ T-cell–mediated antitumor immunity (35, 59, 67, 70). In addition, LPG has been reported to influence γδ T-cell responses and inflammatory cytokine production through TLR2-, TLR4-, and NOD2-associated signaling pathways, thereby contributing to broader adaptive immune regulation (35, 59, 70, 71). Structural variability of LPG among different *Leishmania* species may further influence its immunomodulatory properties, suggesting that distinct LPG variants could differentially shape host immune responses (35, 67, 72, 73).

Overall, PDMs contribute to adaptive immune modulation by influencing DC maturation, shaping antigen presentation, and promoting T-cell differentiation toward effector phenotypes associated with tumor immune control (2, 4, 14, 67). Through coordinated regulation of CD8^+^ T-cell responses and Th1-associated CD4^+^ T-cell polarization, these molecules may help counteract tumor-induced immune suppression and provide a mechanistic basis for parasite-derived immunomodulatory strategies in cancer therapy (2, 13, 14, 67).

### 4.3 TME remodeling

PDMs orchestrate profound remodeling of the TME through coordinated regulation of immune cell composition, cytokine networks, and tumor vascular architecture, thereby shifting the balance from an immunosuppressive to an immune-responsive state (2–4).

A key feature of this remodeling is the reprogramming of the immune cellular landscape, characterized by reduced immunosuppressive cell populations, including Tregs and myeloid-derived suppressor cells (MDSCs), together with enhanced infiltration and activation of cytotoxic immune effector cells, including CD8^+^ T lymphocytes and NK cells (2, 44, 74–76). These coordinated changes contribute to attenuation of tumor-induced immune tolerance and establishment of a microenvironment that supports immune-mediated tumor control (3, 4).

At the molecular signaling level, parasite-derived factors modulate key oncogenic and immunoregulatory pathways within the TME. A representative example is *Toxoplasma gondii* rhoptry protein 18 (ROP18), which has been reported to interfere with STAT3-associated signaling networks implicated in tumor immune evasion (13, 25). Modulation of this pathway is associated with reduced expression of immunosuppressive mediators such as interleukin-10 (IL-10) and transforming growth factor-β (TGF-β), thereby contributing to a shift toward a more pro-inflammatory tumor milieu (2, 25). In parallel, parasite-driven regulation of T-cell–associated cytokine networks further supports the attenuation of Treg-associated immunosuppressive programs within the TME (44, 76).

Beyond immune cell reprogramming, parasite infections also reshape tumor-associated vascular and stromal compartments. *Plasmodium* infection has been shown to modulate tumor-associated macrophage activity and suppress pathological angiogenesis, resulting in partial normalization of tumor vasculature (18, 32). This vascular remodeling is associated with improved perfusion and reduced hypoxia, which together create a microenvironment more permissive for immune cell infiltration and antitumor immune activity (18, 32). Importantly, vascular normalization is increasingly recognized as a key determinant of effective immunotherapy response, as it facilitates immune cell trafficking into tumor tissues and alleviates hypoxia-driven immune exclusion (18, 32).

These findings collectively indicate that PDMs remodel the TME through integrated regulation of immune suppression, effector immune activation, inflammatory signaling networks, and tumor vascular architecture (2, 4). Rather than acting through a single dominant pathway, these effects converge to reprogram the tumor ecosystem toward a state that favors immune surveillance and tumor control (2, 3). This multi-layered reprogramming provides a strong mechanistic rationale for exploring PDMs as modulators of the TME in cancer immunotherapy (2, 4, 13, 14).

## 5 Non-immune antitumor mechanisms

### 5.1 Induction of tumor cell apoptosis and autophagy

PDMs exert direct antitumor effects by inducing programmed tumor cell death through apoptosis and autophagy, two complementary processes that are frequently dysregulated during tumor development (2–4). Restoration of apoptotic signaling is particularly important for eliminating malignant cells that evade physiological cell death (2, 4). PDMs promote apoptosis by regulating the balance between pro- and anti-apoptotic proteins, including Bax and Bcl-2, activating caspase-dependent signaling, and suppressing oncogenic survival pathways (10, 11, 17). In parallel, activation of autophagy further disrupts tumor cell homeostasis, thereby enhancing the cytotoxic effects of PDMs (4, 16).

Among helminth-derived molecules, ES products from *Trichinella spiralis* represent one of the best-characterized examples. These molecules induce apoptosis in multiple tumor models while simultaneously activating AMP-activated protein kinase (AMPK) and suppressing mammalian target of rapamycin (mTOR) signaling, thereby promoting autophagic flux (10, 11, 77, 78). AMPK-mediated inhibition of mTOR facilitates degradation of damaged organelles and proteins, ultimately disrupting tumor cell homeostasis and sensitizing cancer cells to apoptosis (10, 11). The coordinated activation of autophagy and apoptosis suggests that *Trichinella spiralis*–derived molecules eliminate tumor cells through complementary mechanisms rather than through a single signaling pathway (11, 77, 78).

*Schistosoma* egg–derived molecules also exhibit direct antitumor activity through modulation of apoptosis-related signaling pathways (16, 45). SEAs, including immunomodulatory egg-derived factors, have been associated with suppression of PI3K/Akt signaling, thereby reducing tumor cell survival and increasing susceptibility to apoptosis (16, 45, 79). Because persistent activation of PI3K/Akt is a hallmark of many malignancies, inhibition of this pathway provides an important mechanism through which PDMs may restore apoptotic sensitivity (16). Transcriptomic analyses further demonstrate that liver-trapped *Schistosoma* eggs exhibit distinct gene expression profiles, including abundant expression of immunomodulatory molecules, highlighting the complex interaction between parasite-derived factors and the local TME (79, 80). Although the precise contribution of these egg-derived molecules to hepatocellular carcinoma remains to be fully elucidated, accumulating evidence indicates that *Schistosoma* egg-derived molecules can regulate apoptosis, autophagy, and oncogenic signaling in a context-dependent manner (16, 79, 81).

**Figure 2:**
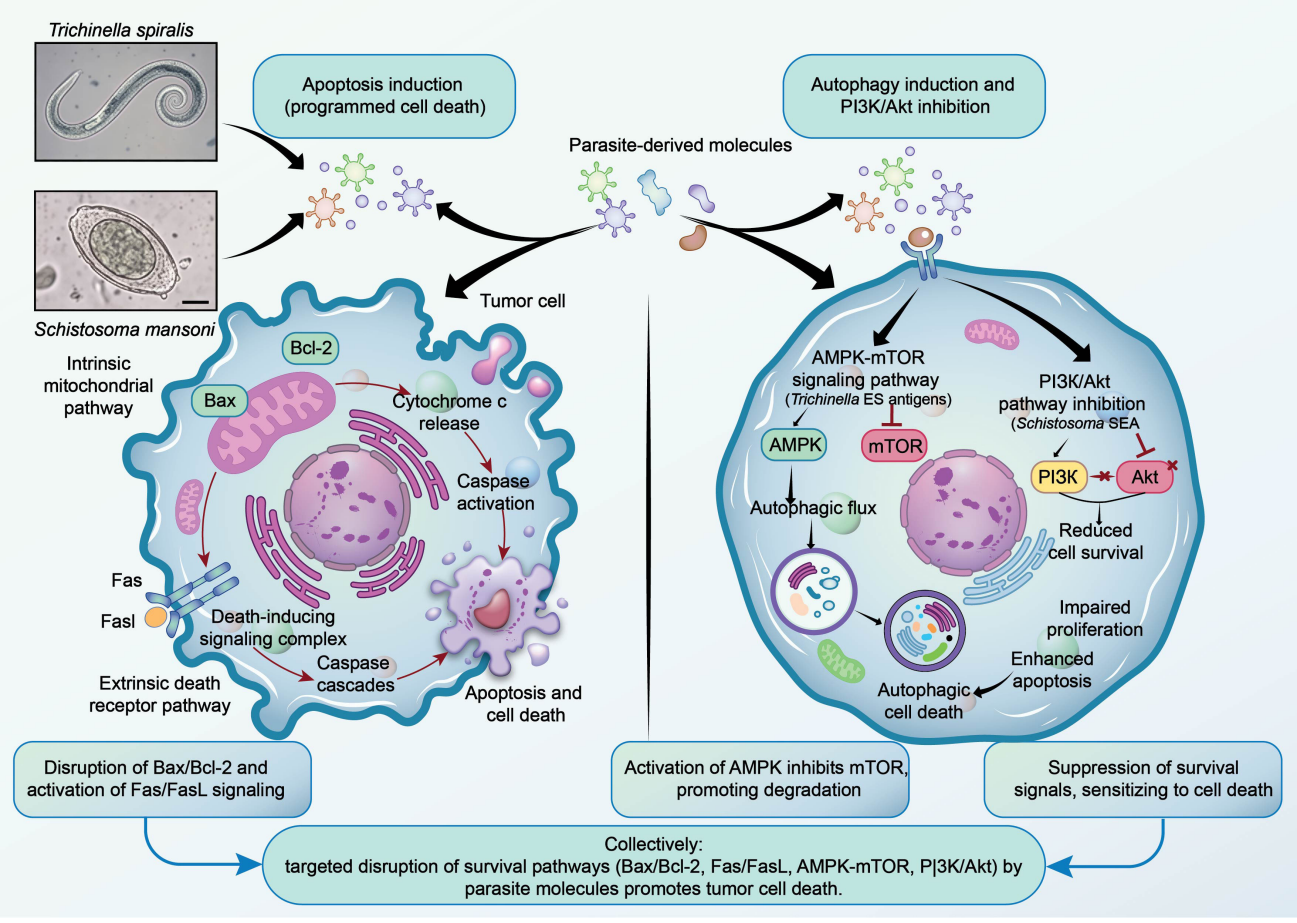
Representative mechanisms of tumor cell apoptosis and autophagy induced by parasite-derived molecules. Parasite-derived molecules induce tumor cell death through coordinated activation of apoptosis and autophagy. *Trichinella spiralis* excretory-secretory (ES) antigens promote apoptosis by regulating Bax/Bcl-2 signaling while activating the AMPK–mTOR pathway to enhance autophagic flux. *Schistosoma* egg-derived molecules, including soluble egg antigens (SEAs) and representative immunomodulatory components such as IPSE/alpha-1, suppress PI3K/Akt signaling, thereby reducing tumor cell survival and increasing susceptibility to apoptosis. Representative findings from hepatocellular carcinoma and colorectal cancer preclinical models illustrate the direct tumoricidal potential of parasite-derived molecules.

Collectively, PDMs directly induce tumor cell death through coordinated activation of apoptosis and autophagy while simultaneously suppressing oncogenic survival pathways (10, 11, 16). These complementary mechanisms provide a strong biological rationale for exploiting PDMs as direct anticancer agents, either alone or in combination with conventional therapies (2–4, 13) (**Figure 2**).

### 5.2 Inhibition of angiogenesis

PDMs inhibit tumor progression by suppressing angiogenesis, a hallmark of cancer that is essential for sustained tumor growth and metastatic dissemination (2–4). These molecules interfere with pro-angiogenic signaling pathways or directly modulate the tumor vasculature, thereby limiting endothelial cell proliferation, migration, and neovascularization (18, 32, 82). Because vascular endothelial growth factor (VEGF) signaling is a major driver of tumor angiogenesis, PDMs that suppress VEGF-associated pathways may effectively deprive tumors of oxygen and nutrients, resulting in impaired tumor growth (18, 32, 83, 84).

Among the best-characterized examples, *Plasmodium* infection has been shown to inhibit tumor angiogenesis by suppressing hypoxia-inducible factor-1α (HIF-1α), a master regulator of hypoxia-driven VEGF expression (32). Downregulation of HIF-1α reduces tumor vascularization and suppresses hepatocellular carcinoma progression, demonstrating that PDMs can modulate hypoxia-responsive signaling pathways that are frequently dysregulated in cancer (32). In addition, *Plasmodium* infection remodels the TME by regulating tumor-associated macrophages, thereby further suppressing angiogenesis and limiting tumor vascular development (18). These complementary mechanisms highlight the ability of PDMs to inhibit angiogenesis through both direct regulation of pro-angiogenic signaling and indirect modulation of the TME (18, 32).

Helminth-derived molecules also exhibit anti-angiogenic potential (16, 85). Recent studies indicate that *Schistosoma* egg-derived molecules, including parasite microRNAs and SEAs, regulate host signaling pathways involved in tumor growth, migration, and angiogenesis (9, 12, 16). Cross-species regulation of host genes by *Schistosoma* microRNAs suppresses hepatocellular carcinoma cell proliferation and migration while inhibiting angiogenesis (9, 12). In particular, *Schistosoma japonicum* sja-let-7 inhibits hepatocellular carcinoma cell growth through cross-species regulation of Col1α2 (85), whereas another *S. japonicum* microRNA suppresses both tumor cell migration and angiogenesis by targeting PGAM1 (9). In addition, *S. japonicum* microRNA targeting FZD4 inhibits hepatoma cell growth and migration, further supporting the anti-angiogenic and antitumor activities of parasite-derived regulatory RNAs (12).

In summary, PDMs suppress tumor angiogenesis through coordinated regulation of hypoxia-responsive pathways, VEGF-associated signaling, tumor-associated macrophages, and parasite-derived regulatory RNAs (12, 18, 32, 82). These complementary mechanisms restrict tumor vascularization and provide a strong mechanistic rationale for developing parasite-derived anti-angiogenic strategies for cancer therapy (2–4, 13).

### 5.3 Inhibition of tumor invasion and metastasis

PDMs inhibit tumor invasion and metastatic progression through coordinated modulation of ECM remodeling, cell–matrix interactions, and tumor cell dissemination processes, which are essential steps in metastatic cascade (2–4). By influencing these biological events, parasite-derived factors are associated with reduced migratory and invasive capacities of tumor cells and contribute to the attenuation of metastatic potential in experimental cancer models (2, 4, 86).

Among *Leishmania*-derived molecules, the surface metalloprotease GP63 has been implicated in host–parasite interaction networks and extracellular matrix regulation (36, 87). GP63 participates in the modulation of ECM-associated proteolytic systems, including matrix metalloproteinases (MMPs), which are key regulators of tissue remodeling and tumor cell invasion (37, 87). Through these interactions, GP63-related activity may influence ECM stability and cell–matrix dynamics, thereby affecting tumor cell motility and invasive behavior (37, 87). In addition, parasite-derived proteases and related effector molecules have been suggested to modulate host signaling pathways involved in tumor–microenvironment communication, further supporting their potential relevance in regulating cancer progression (14, 15, 33).

*Plasmodium falciparum* erythrocyte membrane protein 1 (PfEMP1), a parasite adhesion molecule involved in host erythrocyte binding, has also been investigated in the context of tumor–endothelial interactions (4, 88). Owing to its strong adhesive properties, PfEMP1 represents a model molecule for studying cell adhesion processes within vascular environments (88, 89). Parasite-derived adhesion systems may provide conceptual insight into mechanisms governing tumor cell adhesion to endothelial surfaces and vascular dissemination, suggesting potential translational relevance for metastasis-associated cell–cell interaction studies (4, 88).

Taken together, PDMs are associated with modulation of tumor invasion and metastasis-related processes through effects on ECM remodeling, protease-associated activity, and cell adhesion dynamics. These multi-level interactions highlight parasite-derived systems as valuable biological models for understanding metastatic regulation and exploring potential strategies for interference with tumor dissemination (2–4, 13).

### 5.4 Regulation of metabolic pathways

PDMs interfere with tumor metabolic reprogramming, a hallmark of cancer that supports sustained proliferation, survival, and therapeutic resistance, by modulating redox balance, lipid metabolism, and key intracellular signaling pathways (2–4). Rather than targeting a single metabolic node, these molecules are associated with coordinated alterations in multiple metabolic networks that collectively contribute to disruption of tumor metabolic fitness (2, 4, 90).

A major mechanism involves regulation of oxidative stress. *Toxoplasma gondii* infection has been reported to induce reactive oxygen species (ROS) accumulation, leading to disruption of cellular redox homeostasis and mitochondrial dysfunction in tumor cells (91, 92). Excessive ROS levels are associated with oxidative damage, impaired mitochondrial integrity, and activation of apoptosis-related pathways, ultimately reducing tumor cell viability and proliferative capacity (91, 92). These findings suggest that parasite-induced oxidative stress is associated with metabolic vulnerability in tumor cells (4).

In parallel, helminth-derived molecules regulate lipid metabolic pathways that are essential for membrane biosynthesis and energy storage in rapidly proliferating cancer cells (93). SEAs from *Schistosoma* spp. have been reported to alter signaling pathways such as PI3K/Akt and MAPK that regulate cancer metabolism (16), whereas *Schistosoma* japonicum infection has been associated with broader reprogramming of host glucose and lipid metabolism (93). Disruption of these pathways is linked to reduced tumor cell proliferation and impaired migratory capacity, highlighting a metabolic route through which PDMs may influence tumor progression (16, 45).

In addition, parasite-derived factors are associated with modulation of intracellular signaling pathways that govern metabolic homeostasis, including PI3K/Akt- and MAPK-related networks (82, 94). These pathways integrate growth factor signaling with glucose and lipid metabolism, suggesting that parasite-mediated modulation of these axes may contribute to broader metabolic reprogramming within the TME (82).

**Figure 3:**
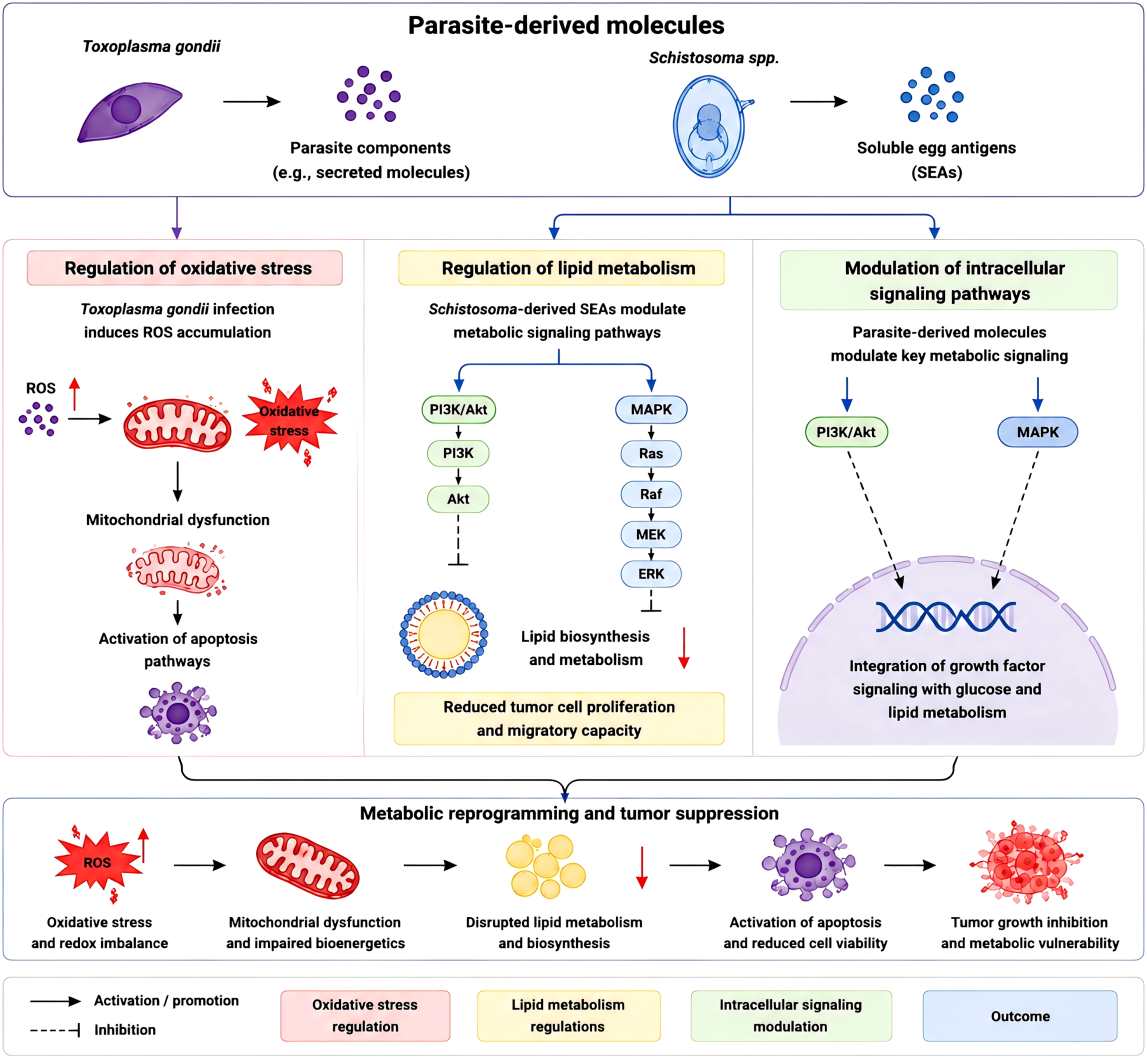
Representative metabolic reprogramming induced by parasite-derived molecules. Parasite-derived molecules regulate tumor metabolic homeostasis through coordinated modulation of oxidative stress, lipid metabolism, and intracellular signaling pathways. *Toxoplasma gondii* infection promotes reactive oxygen species (ROS) accumulation, leading to oxidative stress, mitochondrial dysfunction, and impaired tumor cell viability. *Schistosoma*-derived soluble egg antigens (SEAs) modulate PI3K/Akt- and MAPK-associated signaling pathways involved in lipid biosynthesis and metabolic homeostasis. These findings collectively indicate parasite-derived molecules disrupt tumor metabolic fitness by altering redox balance, lipid metabolism, and metabolic signaling networks, ultimately contributing to metabolic vulnerability and suppression of tumor progression.

In summary, PDMs influence tumor metabolic reprogramming through coordinated effects on oxidative stress regulation (91, 92), lipid metabolism (16, 45), and intracellular metabolic signaling networks (82, 94). These multi-layered interactions suggest that metabolic vulnerabilities represent an additional non-immune axis through which PDMs may suppress tumor progression and provide a rationale for exploring their translational potential as modulators of cancer metabolism (2–4, 13) (**Figure 3**).

## 6 Research progress of parasite-derived molecules in specific cancer types

**Figure 4:**
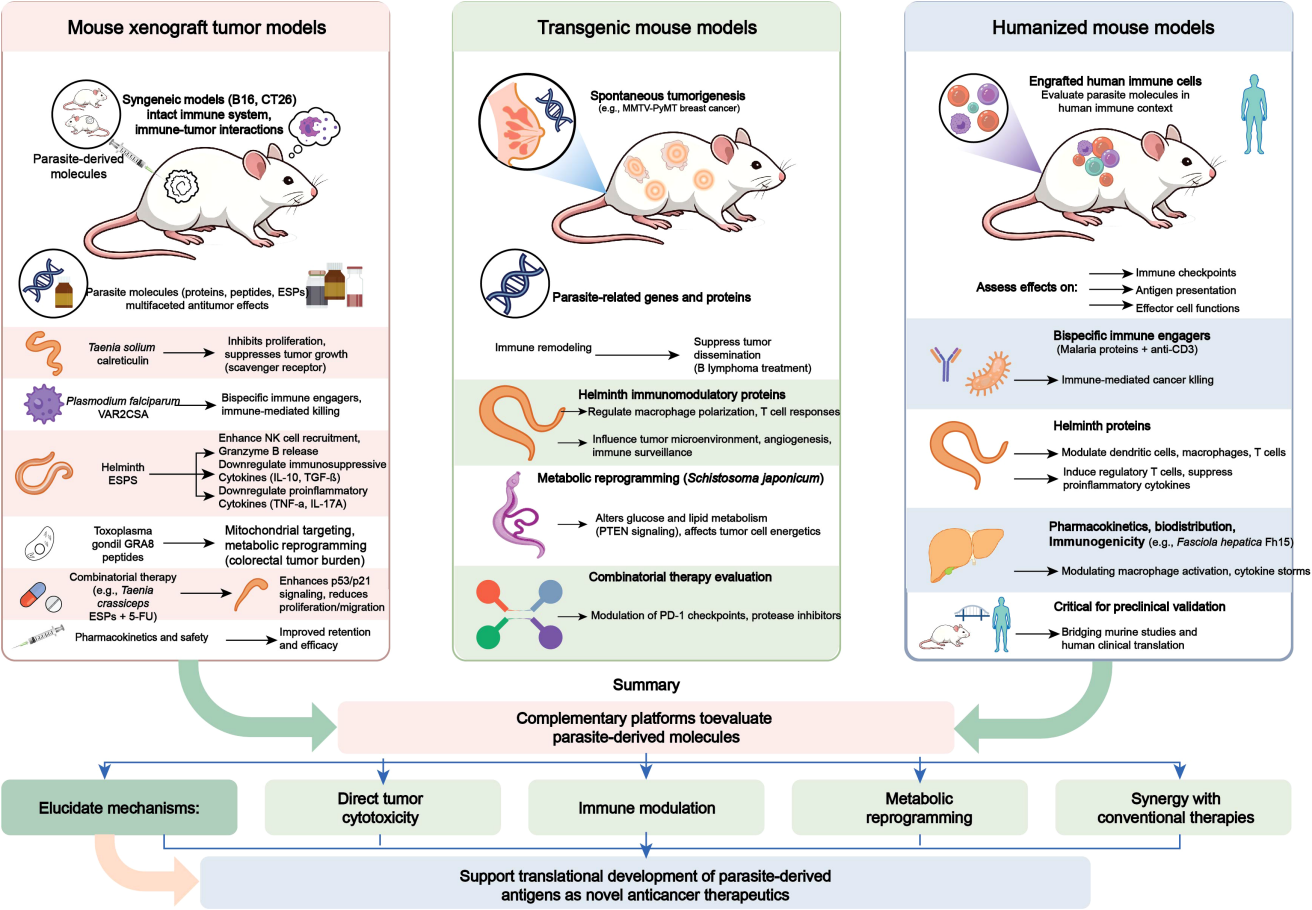
Preclinical *in-vivo* models for evaluating parasite-derived molecules in cancer research. Mouse syngeneic tumor models, genetically engineered mouse models, and humanized mouse models represent complementary preclinical platforms for evaluating the antitumor efficacy and translational potential of parasite-derived molecules. Syngeneic models enable investigation of immune–tumor interactions and direct antitumor effects in immunocompetent hosts. Genetically engineered mouse models facilitate mechanistic studies of tumor initiation, progression, and metabolic reprogramming. Humanized mouse models provide a platform for evaluating parasite-derived molecules in the context of the human immune system and support translational assessment of immunotherapy, pharmacokinetics, immunogenicity, and combination treatment strategies.

Representative preclinical animal models discussed in this section are summarized in **Figure 4**.

### 6.1 Melanoma

Melanoma is one of the most extensively studied tumor models for evaluating the antitumor potential of PDMs, owing to its high immunogenicity and aggressive metastatic behavior, which make it suitable for investigating immune–tumor interactions *in vivo* (2–4). Among preclinical tumor systems, the syngeneic B16 melanoma model has been widely used to evaluate parasite-derived interventions in immunocompetent hosts, enabling assessment of both tumor growth modulation and host immune responses (19, 28, 95). In summary, these models provide a robust experimental platform for investigating how PDMs influence tumor progression through immune-mediated and tumor-intrinsic mechanisms (2–4).

Accumulating preclinical evidence indicates that PDMs exert antitumor effects in melanoma through coordinated regulation of immune activation, TME remodeling, and angiogenesis-associated pathways (4, 13). Among protozoan-derived agents, *Toxoplasma gondii*–derived products have demonstrated consistent antitumor activity in B16 melanoma models, including suppression of tumor growth, prolongation of survival, and reduction of metastatic dissemination, which are associated with enhanced activation of melanoma-reactive CD8^+^ cytotoxic T cells (28). These findings suggest that PDMs may contribute to tumor control through enhancement of effective T-cell–mediated immune responses against melanoma cells (96).

Similarly, *Neospora caninum* has been reported to reduce melanoma metastasis in murine models through activation of innate and adaptive immune responses, including increased CD8^+^ T-cell activity and elevated pro-inflammatory cytokine production (19). These observations further support the role of protozoan-induced immune activation in suppressing melanoma progression in preclinical settings (19).

In addition to protozoan-derived organisms, helminth-derived molecules have also been implicated in melanoma regulation. Cathepsin L1 from *Fasciola hepatica* has been associated with modulation of melanoma cell phenotypic characteristics, suggesting potential involvement in tumor cell reprogramming and tumor–microenvironment interactions (95). Additional evidence indicates that PDMs may also influence melanoma progression through regulation of angiogenesis and tumor microenvironmental signaling pathways (2, 4).

Although preclinical evidence is encouraging, clinical translation remains limited. Current data do not demonstrate clinical evaluation of *Toxoplasma gondii*–derived products in melanoma patients, indicating that this field remains at an early translational stage (13). Major translational barriers include safety evaluation, dosing standardization, immunogenicity control, and limitations in human–model extrapolation, all of which require further investigation before clinical application can be considered (14).

Overall, current evidence suggests that PDMs may regulate melanoma progression through multiple complementary mechanisms (4), including activation of CD8^+^ T-cell–mediated immunity (28, 96), enhancement of innate immune responses (19), modulation of angiogenesis (95), and remodeling of the TME (2, 3, 13). These findings provide a strong preclinical foundation for exploring PDMs as immunomodulatory tools in melanoma therapy and support their potential integration with emerging cancer immunotherapeutic strategies (14).

### 6.2 Lung cancer

**Figure 5:**
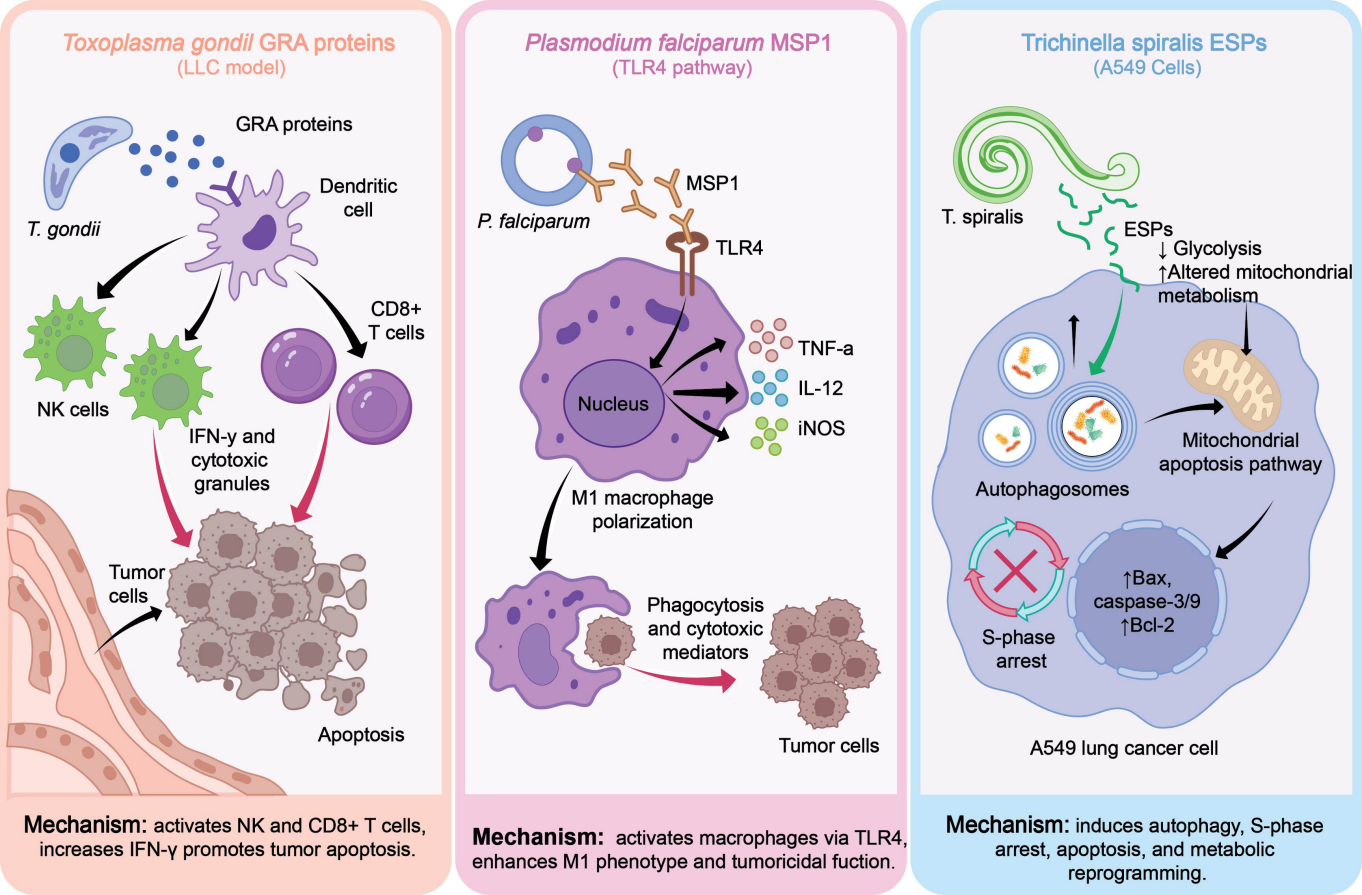
Parasite-derived molecules modulate lung cancer progression. *Toxoplasma gondii* GRA proteins promote activation of natural killer (NK) cells and CD8^+^ cytotoxic T lymphocytes in Lewis lung carcinoma models. This is associated with increased interferon-γ (IFN-γ) production and induction of tumor cell apoptosis. *Plasmodium falciparum* MSP1 is associated with macrophage activation and pro-inflammatory polarization through Toll-like receptor (TLR)–mediated signaling, contributing to enhanced antitumor immune responses. *Trichinella spiralis* excretory/secretory antigens induce autophagy, metabolic stress responses, and apoptotic processes in A549 lung cancer cells, which collectively contribute to inhibition of tumor cell proliferation.

Lung cancer remains one of the leading causes of cancer-related mortality worldwide and continues to present major therapeutic challenges due to its immunosuppressive TME and frequent development of therapeutic resistance (2–4). Recent studies indicate that PDMs may exert antitumor effects in lung cancer through both immune-mediated mechanisms and tumor-intrinsic regulatory processes, including apoptosis, autophagy, metabolic reprogramming, and angiogenesis-associated pathways (11). Preclinical studies using Lewis lung carcinoma models and human non-small cell lung cancer (NSCLC) cell lines, particularly A549 cells, have consistently demonstrated that parasite-derived products may suppress tumor progression through coordinated immune activation and direct tumor cell regulation (10, 48, 77, 97) (**Figure 5**).

Accumulating experimental evidence indicates that protozoan-derived molecules may enhance antitumor immune responses in lung cancer models (13). *Toxoplasma gondii* dense granule (GRA) proteins have been shown to activate innate and adaptive immune responses in Lewis lung carcinoma models (97). These effects are associated with increased infiltration of NK cells and CD8^+^ cytotoxic T lymphocytes, accompanied by elevated IFN-γ production and enhanced tumor cell apoptosis (97). These findings suggest that PDMs may contribute to tumor growth suppression through coordinated activation of antitumor immune responses (13).

Similarly, *Plasmodium falciparum* merozoite surface protein 1 (MSP1) has been reported to modulate macrophage-mediated immune responses through Toll-like receptor-associated signaling pathways (18). MSP1 is associated with macrophage polarization toward a pro-inflammatory phenotype, leading to increased cytokine secretion and enhanced tumoricidal activity (18). These activated macrophages may further facilitate recruitment of additional immune effector cells, thereby contributing to a sustained pro-inflammatory TME that is less permissive to tumor progression (32).

In addition to immune regulation, PDMs also exert direct effects on lung cancer cells. *Fasciola* hepatica ESPs reduce A549 cell viability and are associated with increased ROS production and induction of apoptotic processes, suggesting disruption of tumor cell redox homeostasis (49). Similarly, *Trichinella spiralis*-derived excretory/secretory products inhibit proliferation of A549 non-small cell lung cancer cells in a dose- and time-dependent manner (10, 11). These effects are associated with activation of autophagy, induction of mitochondrial apoptosis, and S-phase cell-cycle arrest (10, 77). Transcriptomic analyses further suggest that metabolic pathways, including glycolysis and the pentose phosphate pathway, may be suppressed, while mitochondrial metabolic activity is altered, indicating parasite-associated metabolic reprogramming in tumor cells (77). In addition, antibody-mediated targeting of *Trichinella spiralis* antigens has been shown to reduce tumor growth and decrease expression of proliferation- and angiogenesis-related markers such as PCNA and VEGF (48). Taken together, these findings suggest that PDMs may regulate lung cancer progression through coordinated modulation of apoptosis, metabolic stress responses, and angiogenesis-related pathways (11).

Although preclinical evidence is encouraging, clinical translation of parasite-derived strategies in lung cancer remains limited, and no clinical trials have yet been reported evaluating parasite-based therapies in lung cancer patients (13). Therefore, current evidence is primarily derived from preclinical models and experimental systems, highlighting a significant translational gap that warrants further investigation to evaluate safety, efficacy, and clinical applicability (14).

Overall, available studies suggest that PDMs may suppress lung cancer progression through multiple complementary mechanisms (4), including activation of innate and adaptive immune responses (13, 97), modulation of macrophage polarization (18, 32), induction of apoptosis and autophagy (10, 11, 49), metabolic reprogramming (77), and regulation of angiogenesis-associated pathways (48). These findings provide a strong preclinical rationale for further exploration of PDMs as potential immunomodulatory and anticancer agents in lung cancer therapy (2, 3, 14).

### 6.3 Colorectal cancer

CRC remains a major global health burden due to its high incidence, frequent metastatic progression, and limited responsiveness to immunotherapy in a substantial subset of patients. Emerging evidence suggests that PDMs may exert antitumor effects in CRC through coordinated regulation of tumor cell proliferation, immune microenvironment remodeling, intracellular signaling pathways, and host–microbiota interactions (2–4).

Both *in-vitro* and *in-vivo* experimental studies indicate that PDMs may regulate CRC progression through multiple complementary mechanisms (3). Antigens derived from gastrointestinal nematodes have been reported to suppress CRC cell proliferation, potentially involving regulation of cell-cycle progression and epithelial–mesenchymal transition-associated pathways (98). In addition, excretory/secretory products from *Ascaris lumbricoides* are associated with reduced viability of HCT116 CRC cells *in vitro*, suggesting potential direct cytotoxic effects of helminth-derived bioactive components (52). Parasite-associated inflammatory environments, including those linked to *Toxoplasma gondii*, have been shown to modulate cytokine networks such as IFN-γ and IL-18, which may indirectly influence proliferation-related signaling in CRC models (13, 46).

In murine CRC models, *Schistosoma mansoni* SEA has been shown to suppress tumor growth in CT26-bearing mice and enhance the therapeutic efficacy of immune checkpoint blockade (99). These effects are associated with remodeling of the tumor immune microenvironment, including reduced regulatory T-cell infiltration, increased CD8^+^ T-cell activation, and elevated pro-inflammatory cytokine production such as IFN-γ (99). These findings suggest that PDMs are capable of overcoming resistance to immunotherapy in microsatellite-stable CRC through immune microenvironment reprogramming (14).

In addition, PDMs may regulate CRC progression through modulation of intracellular signaling pathways. *Toxoplasma gondii* rhoptry protein 18 (ROP18) has been reported to influence STAT3-associated signaling networks, which are closely linked to tumor immune evasion and inflammatory regulation (100). Such modulation may contribute to attenuation of immunosuppressive cytokine signaling and support antitumor immune activation in CRC contexts (13, 100).

Emerging evidence further suggests that parasite–host interactions may influence CRC progression through gut microbiota modulation (9). Parasite-associated environments have been associated with shifts in intestinal microbial composition, including enrichment of short-chain fatty acid (SCFA)–producing bacteria (85). Metabolites such as butyrate and propionate are associated with suppression of chronic inflammation, maintenance of intestinal epithelial barrier integrity, and regulation of tumor-associated immune responses (9, 85). Consistently, sodium butyrate has been shown to modulate immune cell populations in CT26 tumor-bearing mice, including reduction of regulatory T cells and increased activation of innate and adaptive immune subsets such as NK and Th17 cells (101).

**Figure 6:**
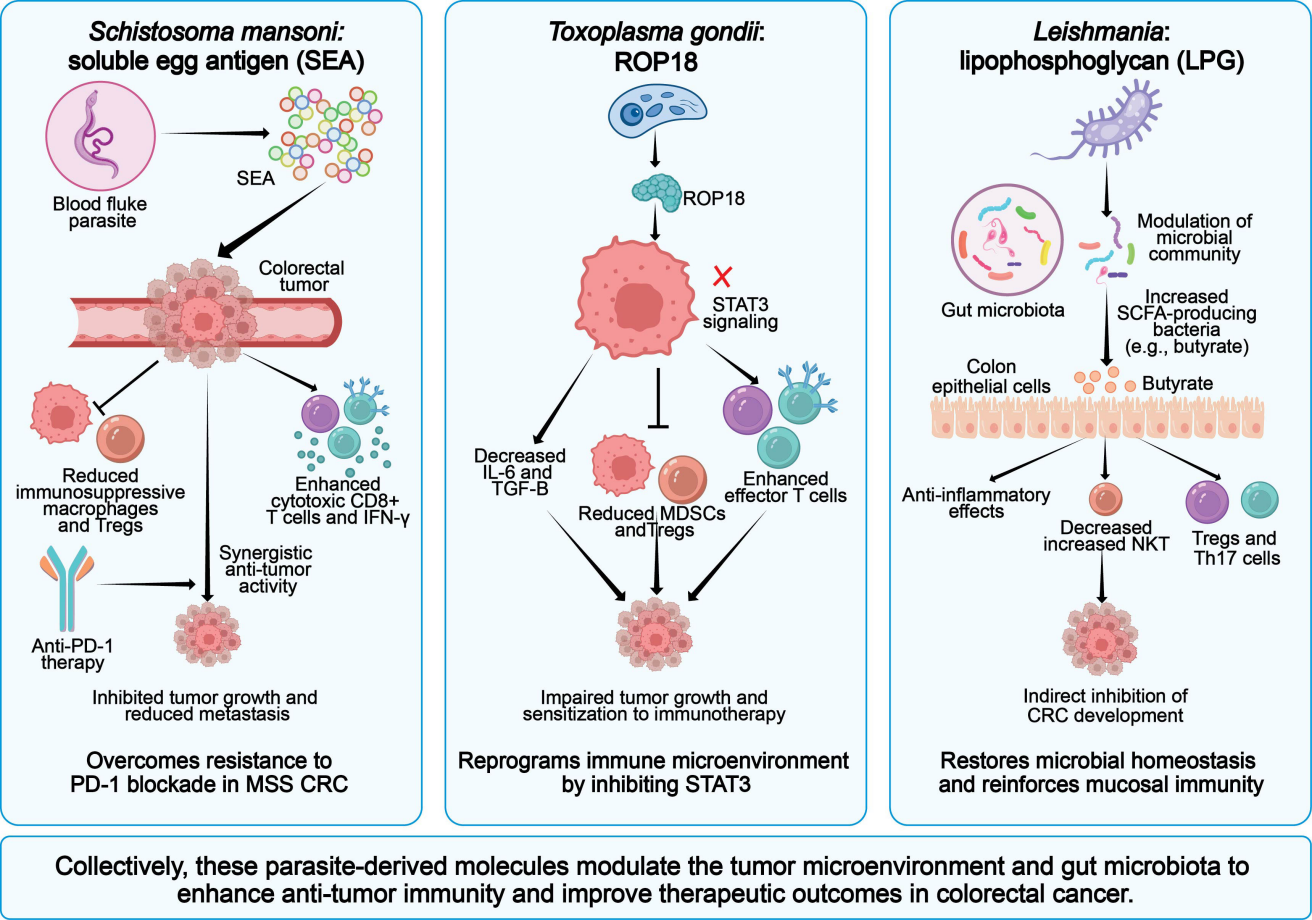
Parasite-derived molecules modulate colorectal cancer progression. *Schistosoma mansoni* soluble egg antigen (SEA) remodels the tumor immune microenvironment in CT26 colorectal cancer models, characterized by reduced regulatory T-cell infiltration, decreased immunosuppressive macrophage activity, enhanced CD8^+^ T-cell activation, and improved responsiveness to immune checkpoint blockade. *Toxoplasma gondii* rhoptry protein 18 (ROP18) is associated with modulation of STAT3-related signaling pathways and downstream inflammatory cytokine networks, contributing to attenuation of immunosuppressive signaling and enhancement of antitumor immune responses in colorectal cancer contexts. Parasite-associated modulation of gut microbiota and host metabolism contributes to colorectal cancer regulation, including enrichment of SCFA-producing bacteria and regulation of immune responses mediated by microbiota-derived metabolites such as butyrate. These effects collectively contribute to immune microenvironment reprogramming and suppression of colorectal cancer progression.

Overall, experimental studies indicate that PDMs may suppress CRC progression through multiple interconnected mechanisms (4), including inhibition of tumor cell proliferation (46, 52, 98), modulation of immune cell infiltration and function and enhancement of immunotherapy responsiveness (13, 14, 99), regulation of STAT3-associated signaling (100), and microbiota-associated metabolic reprogramming (9, 85, 101). These findings provide a strong preclinical rationale for further exploration of PDMs as immunomodulatory agents or therapeutic adjuvants for CRC (2, 3) (**Figure 6**).

### 6.4 Breast cancer

Breast cancer remains one of the leading causes of cancer-related morbidity and mortality worldwide, with triple-negative breast cancer (TNBC) representing a particularly aggressive subtype lacking effective targeted therapeutic options. Increasing evidence suggests that PDMs may exert antitumor effects in breast cancer through coordinated regulation of immune responses, tumor cell proliferation, metastasis-related processes, ECM remodeling, and treatment sensitivity (2, 4, 14).

*In vitro* studies suggest that parasite-derived or parasite-inspired molecules may directly influence breast cancer cell viability. Organometallic complexes incorporating artemisinin derivatives, including rhodium- and iridium-based compounds, have been reported to exhibit cytotoxic activity against MCF-7 breast cancer cells, suggesting potential anticancer properties of parasite-derived chemical scaffolds (102). In addition, recombinant calreticulin derived from parasitic organisms has been shown to suppress breast cancer cell proliferation and colony formation, indicating potential involvement in tumor growth regulation, although the precise mechanisms in breast cancer contexts remain to be fully clarified (103).

*In vivo* evidence from genetically engineered and syngeneic mouse models, including the MMTV-PyMT transgenic model (3), suggests that parasite-associated immunomodulatory factors may influence tumor progression through regulation of macrophage polarization, T-cell responses, and TME remodeling, thereby contributing to modulation of tumor growth and metastatic dissemination (74).

Among protozoan-derived systems, attenuated *Toxoplasma gondii* strains have demonstrated consistent antitumor activity in the 4T1 murine breast cancer model (13, 20, 29). Infection with avirulent strains is associated with reduced tumor growth and lung metastasis, accompanied by enhanced Th1-type immune responses, including increased production of IL-12 and IFN-γ (20, 29). These effects are further associated with increased infiltration of immune effector cells, including NK cells, dendritic cells, macrophages, and T lymphocytes, suggesting reprogramming of the tumor immune microenvironment toward a more immunostimulatory state (13).

Similarly, *Plasmodium falciparum*–derived molecules have been reported to modulate breast cancer metastasis and immune responses (74, 104). Parasite-derived adhesion-related molecules may interfere with tumor–endothelial interactions, potentially contributing to reduced metastatic dissemination (105). In addition, *Plasmodium*-associated immune activation has been linked to increased CD8^+^ T-cell responses, including enhanced cytotoxic activity and granzyme B expression (104), which may contribute to suppression of tumor progression in murine models (74).

Parasite-associated protease systems, particularly cathepsin L-related pathways, have also been implicated in breast cancer progression. These systems and related probes have been used to investigate tumor invasion and chemoresistance, especially in aggressive breast cancer subtypes such as MCF-7/ADR cells, suggesting a potential role in extracellular matrix remodeling and invasion-associated processes (106–108).

Although preclinical evidence is promising, whether these findings can be reproduced clinically remains unclear (13). Therefore, current evidence is primarily derived from experimental models, and further translational studies are required to evaluate safety, efficacy, and clinical feasibility (14).

**Figure 7:**
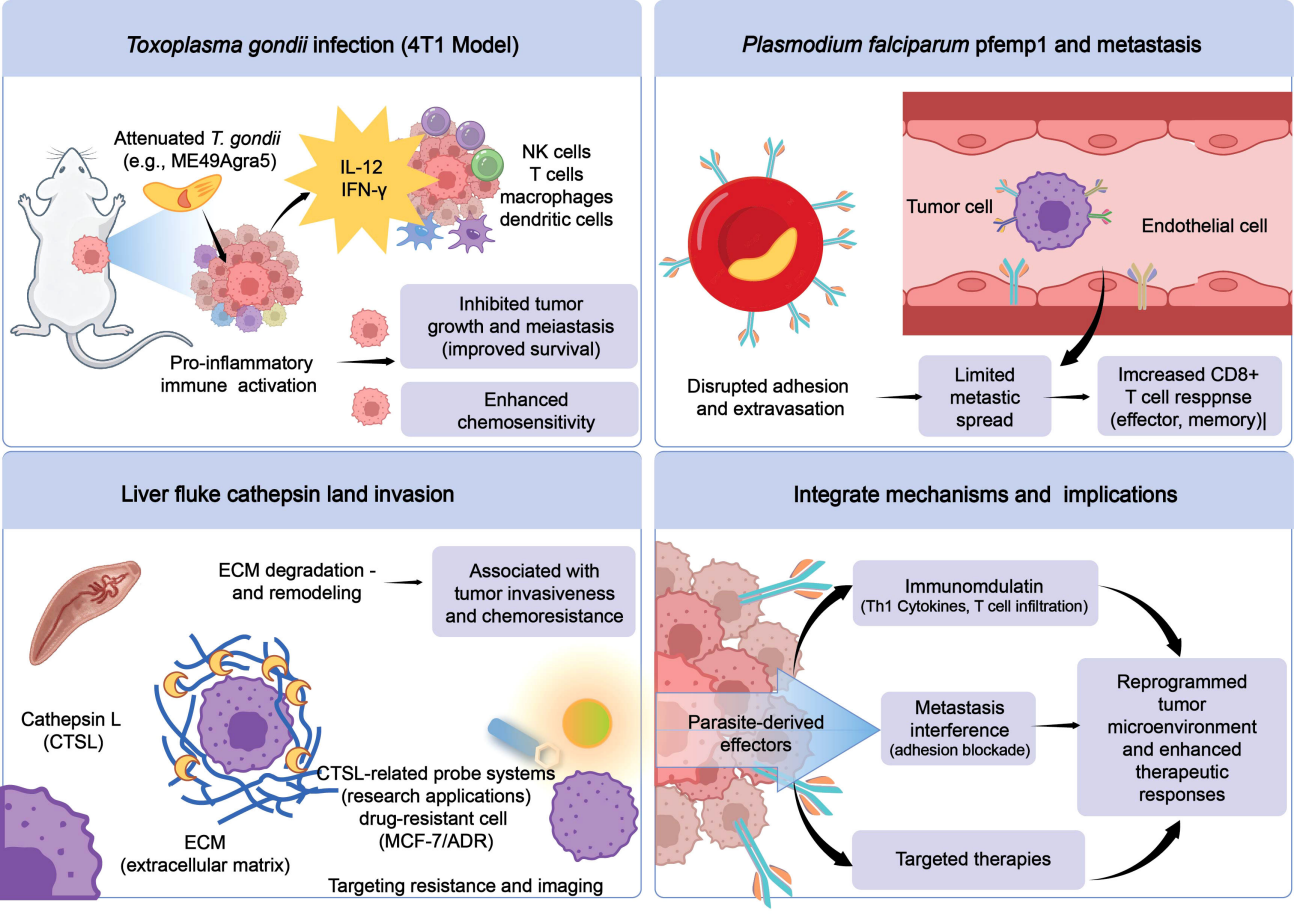
Parasite-derived molecules modulate breast cancer progression and metastasis. Attenuated *Toxoplasma gondii* strains are associated with induction of Th1-type immune responses and activation of cytotoxic T cells, contributing to reduced tumor growth and metastasis in 4T1 breast cancer models. *Plasmodium falciparum* PfEMP1 is involved in modulation of tumor–endothelial interactions, which may contribute to reduced metastatic dissemination. Liver fluke-associated cathepsin L-related pathways are linked to extracellular matrix remodeling and regulation of tumor cell invasiveness and treatment resistance. Collectively, these parasite-associated mechanisms contribute to remodeling of the TME, suppression of metastatic dissemination, and modulation of breast cancer progression.

Overall, recent investigations demonstrate that PDMs may influence breast cancer progression through multiple complementary mechanisms (4), including modulation of Th1-type immune responses, regulation of tumor–immune cell interactions, interference with tumor–endothelial adhesion, and remodeling of extracellular matrix-related pathways. These findings provide a strong preclinical foundation for further exploration of PDMs as immunomodulatory and anticancer agents in breast cancer therapy (2, 14) (**Figure 7**).

### 6.5 Liver cancer

Liver cancer remains one of the most lethal malignancies worldwide due to its high metastatic potential, complex immunosuppressive TME, and limited responsiveness to current therapeutic strategies. Experimental evidence demonstrates that PDMs may exert antitumor effects in liver cancer through coordinated regulation of immune responses, angiogenesis, tumor cell survival, and metabolic pathways (15, 93).

Accumulating preclinical evidence indicates that *Plasmodium* infection may suppress tumor progression in the H22 murine hepatoma model (18). These effects are associated with reduced tumor growth and prolonged survival, potentially involving modulation of tumor angiogenesis and enhancement of antitumor immune responses (32). Parasite-derived hemozoin accumulation in tumor-associated macrophages has been linked to regulation of IGF-1–related signaling and downstream PI3K/MAPK pathways, which may contribute to decreased expression of angiogenesis-related molecules such as MMP-9 (18). In addition, suppression of hypoxia-associated signaling, including reduced HIF-1α expression, has been observed in *Plasmodium*-associated tumor models, which may further contribute to impaired tumor vascularization (32). Enhanced infiltration of CD8^+^ T cells and increased IFN-γ production have also been associated with a shift toward Th1-type immune responses, suggesting immune-related suppression of hepatoma progression (18).

Schistosome-derived non-coding RNAs, particularly microRNAs from *Schistosoma* japonicum, have been implicated in the regulation of hepatocellular carcinoma progression (85). These molecules are associated with modulation of host gene expression networks, including targets such as FZD4, PGAM1, and Col1α2, which are involved in tumor proliferation, migration, and angiogenesis (12). These findings suggest that parasite-derived regulatory RNAs may participate in cross-species gene regulation and contribute to modulation of tumor-associated signaling pathways in liver cancer (9).

In addition to immune-related mechanisms, PDMs may also exert direct inhibitory effects on hepatocellular carcinoma cells (78). Helminth-derived peptides identified through proteomic analyses have been associated with suppression of tumor cell proliferation and induction of metabolic stress responses. Furthermore, *Leishmania* donovani has been reported to induce a pro-inflammatory TME in hepatocellular carcinoma, which may contribute to enhanced antitumor immune activity and suppression of tumor growth (15).

Experimental models of parasitic infection further support the involvement of parasite–host interactions in liver tumor regulation. *Echinococcus multilocularis* infection has been associated with reduced hepatic inflammation and modulation of endoplasmic reticulum stress-related pathways in the liver (109). In addition, *Schistosoma* japonicum infection has been linked to metabolic reprogramming of hepatic glucose and lipid metabolism, suggesting that parasite–host interactions may influence the metabolic landscape of the liver TME (93).

Although preclinical evidence is encouraging, further validation in clinically relevant models remains necessary. Therefore, current evidence is primarily derived from experimental models, and further translational studies are required to evaluate safety, efficacy, and clinical feasibility (15, 18).

Taken together, preclinical evidence has shown that PDMs may suppress liver cancer progression through multiple complementary mechanisms, including modulation of immune responses, inhibition of angiogenesis, regulation of hypoxia-associated signaling, induction of tumor cell stress responses, and remodeling of hepatic metabolic pathways (9, 12, 32, 78, 85). These findings provide a strong preclinical rationale for further investigation of PDMs as potential immunomodulatory and metabolic regulators in liver cancer therapy.

### 6.6 Other solid tumors and hematological malignancies

Although research in specific tumor types remains limited within this subsection, PDMs have increasingly been recognized as immunomodulatory agents capable of influencing tumor-associated immune responses, inflammation, and host–parasite interaction networks (2, 3, 110). These effects suggest broad relevance in experimental oncology models (4).

In ovarian cancer models, recombinant *Taenia solium* calreticulin has been shown to inhibit proliferation and colony formation of cancer stem-like cells *in vitro* (103). These findings further support the role of parasite-derived immunomodulatory proteins in regulating tumor cell viability through host-associated immune mechanisms (4, 103).

In hematological malignancy-related systems, parasite-induced host reprogramming has been observed in *Theileria annulata*–transformed macrophages, where tumor-associated gene signatures such as GZMA and RASGRP1 suggest regulation of malignant transformation and immune-associated tumor control (3, 23).

More broadly, PDMs, including excretory/secretory products and glycoconjugates, have been reported to modulate macrophage activation and T-cell differentiation, thereby contributing to immune microenvironment remodeling in experimental disease models (44, 54, 76, 111). These regulatory effects are further supported by evidence indicating parasite-associated modulation of inflammatory signaling pathways (64).

In addition, parasite-derived systems have been increasingly explored as experimental immunotherapeutic platforms. Recombinant parasite-associated molecules and engineered immune modulators have demonstrated the ability to activate or redirect host immune responses, highlighting their potential as biologically derived immunotherapy scaffolds (4, 105, 112).

Currently, no clinical studies have evaluated parasite-derived therapeutic strategies in ovarian cancer or hematological malignancies (3, 110). Existing evidence remains entirely derived from *in vitro* systems, animal models, and mechanistic immunology studies, indicating an early translational stage of development (3, 4).

In summary, available studies indicate that PDMs exhibit broad immunomodulatory and tumor-regulatory potential in experimental systems, including suppression of tumor cell proliferation, modulation of immune cell function, and regulation of host immune signaling networks (2, 23, 103). However, these effects remain preclinical in nature, and further translational validation is required to determine their therapeutic potential in cancer treatment (3, 4, 110).

## 7 Translational potential and future perspectives

### 7.1 Combination therapeutic strategies

Combination therapy has become an important strategy for improving the efficacy of cancer treatment by overcoming tumor immune evasion, therapeutic resistance, and the immunosuppressive TME. Owing to their immunomodulatory properties, PDMs have attracted increasing attention as adjunctive agents capable of enhancing the efficacy of existing anticancer therapies (3, 4). Overall, these observations suggest that these molecules may potentiate immune checkpoint blockade, improve responses to conventional chemotherapy, and modulate treatment-related tissue responses, highlighting their translational potential in combination strategies (14, 66, 97, 113).

Among the currently investigated approaches, combination with immune checkpoint inhibitors has demonstrated promising preclinical evidence. In the CT26 CRC model, *Schistosoma mansoni* SEA enhanced the efficacy of anti–PD-1 therapy by remodeling the tumor immune microenvironment, reducing Tregs and immunosuppressive macrophages, and promoting CD8^+^ T-cell activation (99). Similarly, experimental *Echinococcus multilocularis* infection has been associated with modulation of the PD-1/PD-L1 axis and reduced hepatic inflammation, supporting the rationale for combining parasite-derived immunomodulation with immune checkpoint blockade (109, 114). More broadly, studies investigating PD-1 signaling in infection and cancer further support immune checkpoint modulation as a promising strategy for parasite-based immunotherapy (115–118).

PDMs may also enhance the efficacy of conventional anticancer therapies. Recombinant *Taenia solium* calreticulin (rTsCRT) has been shown to synergize with 5-fluorouracil, resulting in enhanced inhibition of tumor cell viability and colony formation (103). Likewise, helminth-derived molecules have been reported to improve the therapeutic efficacy of 5-fluorouracil in experimental colorectal tumorigenesis (47). Although direct experimental evidence remains limited, parasite-derived immunomodulators may enhance radiotherapy-induced immunogenic cell death, promote abscopal responses, and reduce radiation-associated tissue injury through regulation of inflammatory and oxidative stress pathways. These hypotheses require further experimental validation.

Taken together, current evidence supports the application of PDMs as adjuncts to existing cancer therapies, particularly immune checkpoint blockade and chemotherapy (2, 13). Although most studies remain at the preclinical stage, these findings provide a strong rationale for further mechanistic and translational investigations.

**Figure 8:**
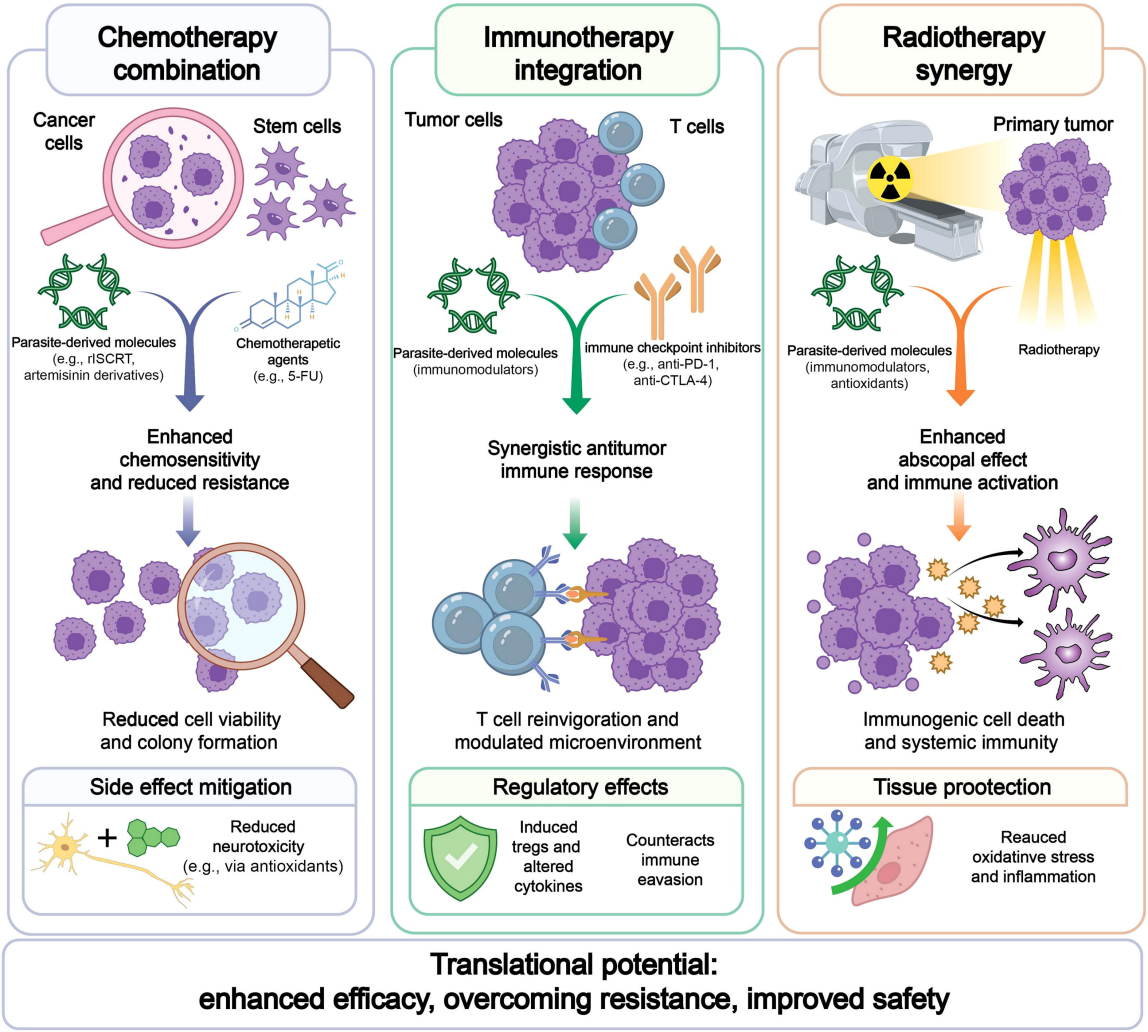
Combined therapeutic strategies using parasite-derived molecules. Parasite-derived molecules may enhance the efficacy of conventional cancer therapies, particularly chemotherapy and immune checkpoint blockade. Recombinant *Taenia solium* calreticulin (rTsCRT) in combination with 5-fluorouracil (5-FU) is associated with enhanced inhibition of tumor cell viability and colony formation. Parasite-derived immunomodulators may enhance immune checkpoint blockade by promoting CD8^+^ T-cell activation and reducing immunosuppressive cell populations within the TME. Although experimental evidence remains limited, parasite-derived molecules may also enhance radiotherapy-induced antitumor immunity and tissue protection. Overall, these combination strategies may improve therapeutic efficacy through coordinated modulation of the tumor immune microenvironment.

Beyond current evidence, PDMs may also be integrated with emerging immunotherapeutic platforms. Their ability to activate APCs, promote Th1 polarization, and enhance CD8^+^ T-cell responses provides a rationale for combination with adoptive cell therapies such as chimeric antigen receptor T cells (119–121). Likewise, their immunostimulatory properties may enhance cancer vaccine efficacy and may be compatible with oncolytic virotherapy and extracellular vesicle-based strategies, although these approaches remain to be experimentally validated (60, 71, 122) (**Figure 8**).

### 7.2 Immunogenicity and safety

Despite their promising antitumor activities, the immunogenic nature of PDMs remains a major challenge for clinical translation. As exogenous bioactive molecules, parasite-derived proteins and antigens may induce excessive immune activation, hypersensitivity, or immune dysregulation, while their long-term immunomodulatory effects remain insufficiently understood (2, 13, 123). Therefore, balancing therapeutic immune activation with long-term safety will be essential for the successful clinical development of parasite-derived therapeutics (2, 3).

One major concern is the potential immunogenicity of PDMs. Because many parasite antigens share structural similarities with host proteins or possess potent immunostimulatory properties, they may elicit hypersensitivity reactions, excessive inflammatory responses, or even autoimmune manifestations following repeated administration (123–126). Although immune activation is fundamental to their antitumor efficacy, excessive or persistent stimulation may compromise treatment safety and reduce therapeutic benefit (2, 3, 13).

Long-term immune modulation also warrants careful consideration. Parasites have evolved sophisticated mechanisms to regulate host immunity, frequently promoting regulatory immune responses that facilitate immune evasion during chronic infection (2, 4, 7). Consequently, prolonged exposure to PDMs may induce immune tolerance or immunosuppressive microenvironments that attenuate durable antitumor immunity (3, 4, 13, 19). Furthermore, persistent inflammatory responses during chronic parasitic infections, particularly those associated with *Leishmania* or *Schistosoma mansoni*, have been implicated in tissue fibrosis and carcinogenesis, highlighting the context-dependent nature of parasite-induced immune regulation (109, 127–130).

Another important safety concern involves off-target biological effects. PDMs that Tregs, MDSCs, or other immune populations may disrupt immune homeostasis, thereby increasing the risk of autoimmune disorders or impairing host defense against opportunistic pathogens (13, 44, 76, 131). In addition, parasite-derived microRNAs have been reported to participate in liver fibrosis and hepatocellular carcinoma progression, suggesting that some parasite-derived products may influence tissue remodeling beyond their intended antitumor activity (85). Therefore, potential organ toxicity, fibrosis, and unintended immunological consequences should be carefully evaluated during preclinical development (123, 125, 126).

Comprehensive safety evaluation should therefore accompany the development of parasite-derived therapeutics throughout the translational process. Preclinical studies should systematically assess acute and chronic toxicity, immunogenicity, biodistribution, off-target effects, and reproductive or genetic toxicity, particularly for live or attenuated parasite-based therapies (125, 126). Long-term clinical follow-up will also be necessary to monitor delayed adverse events, including chronic inflammation, autoimmunity, secondary malignancies, and infection susceptibility associated with immune modulation (117, 118). Moreover, the development of sensitive biomarkers capable of detecting early immune dysregulation and tissue injury may facilitate patient stratification, safety monitoring, and individualized treatment optimization (62, 117, 132).

Overall, although PDMs have demonstrated encouraging therapeutic potential, their successful clinical translation will depend on rigorous safety assessment and careful control of immunogenicity (3, 133, 134). Future studies should focus on optimizing the balance between antitumor immune activation and long-term safety while establishing standardized preclinical and clinical evaluation systems to support their safe application in cancer therapy (13, 125, 126).

### 7.3 Delivery system optimization

The successful clinical translation of PDMs largely depends on the development of efficient delivery systems capable of preserving their biological activity while improving pharmacokinetic properties (135, 136). Because most parasite-derived therapeutics are proteins, peptides, or other macromolecules, they are susceptible to enzymatic degradation, rapid systemic clearance, and limited tumor accumulation, all of which may substantially compromise therapeutic efficacy. Therefore, optimizing delivery strategies has become an essential step toward maximizing their clinical potential (122).

Nanotechnology-based delivery platforms provide effective solutions for improving the stability and bioavailability of PDMs (41, 122). Liposomes can encapsulate both hydrophilic and hydrophobic molecules, protecting them from proteolytic degradation while facilitating cellular uptake. Likewise, polymeric and lipid nanoparticles offer additional advantages, including controlled drug release, improved pharmacokinetic profiles, and surface functionalization for active tumor targeting. Recent advances in lipid nanoparticle (LNP)–based systems, particularly mRNA-LNP platforms, have further expanded the feasibility of delivering parasite- or pathogen-derived antigens for cancer immunotherapy (119). Viral vectors represent another promising approach by enabling sustained intracellular expression of parasite-derived therapeutic molecules within tumor tissues (60, 135).

Targeted delivery further improves therapeutic specificity while minimizing systemic toxicity (105). Conjugation of PDMs or their carriers with tumor-specific antibodies or receptor-targeting ligands enhances selective accumulation within tumor tissues. Such strategies not only increase local drug concentrations but also improve the therapeutic index of parasite-derived therapeutics (137). Advances in antibody engineering and immune engager platforms derived from pathogen-associated molecules further support this targeting concept (119).

Controlled-release systems provide an additional strategy to prolong therapeutic activity *in vivo* (122, 136, 138). Hydrogels and biodegradable microspheres can maintain sustained release of encapsulated PDMs, thereby extending their biological half-life and reducing the frequency of administration (122, 136, 139). These systems are particularly attractive for immunomodulatory therapies, where prolonged and controlled antigen exposure may facilitate durable immune activation while minimizing fluctuations in drug concentration (68, 71, 119).

Beyond improving pharmacokinetics, delivery systems should also preserve the structural integrity and immunological activity of PDMs. Co-delivery of parasite antigens together with adjuvants or other immunostimulatory agents may further enhance antigen presentation and promote robust antitumor immune responses (28, 71). For example, optimized delivery platforms have been proposed to improve the prophylactic antitumor efficacy of *Toxoplasma gondii* antigens by prolonging antigen presentation and sustaining immune activation (60, 119, 122).

**Figure 9:**
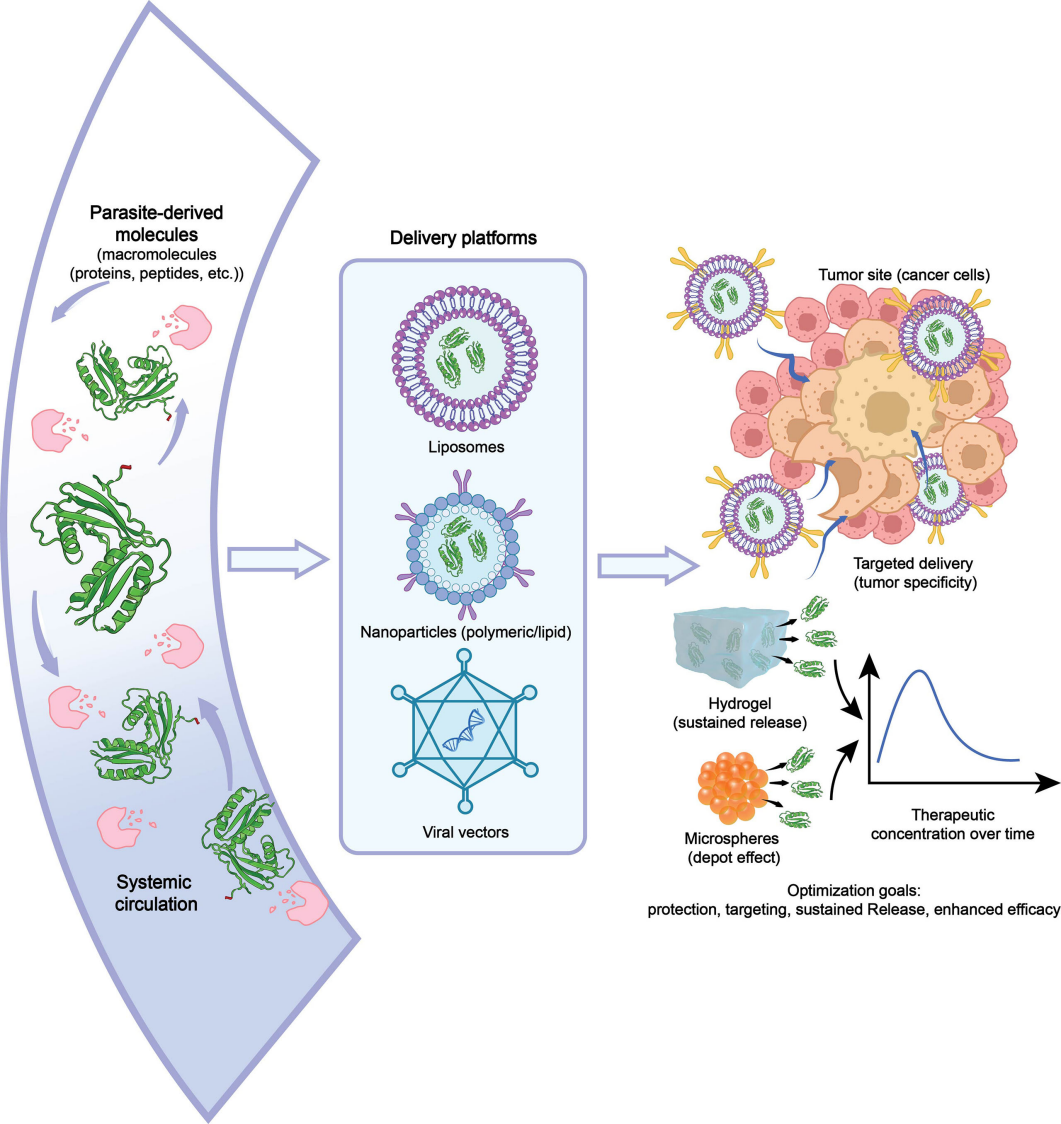
Delivery system optimization for parasite-derived molecules. Parasite-derived macromolecules are susceptible to enzymatic degradation and exhibit limited bioavailability. Liposomes, nanoparticles, viral vectors, hydrogels, and microspheres can enhance molecular stability, protect bioactivity, and enable sustained release. These delivery systems facilitate improved pharmacokinetic profiles and support controlled *in vivo* release of parasite-derived therapeutics. Collectively, nanocarriers and controlled-release platforms contribute to improved tumor delivery efficiency and enhanced therapeutic performance in cancer models.

In summary, advances in delivery technologies provide important opportunities to overcome one of the major translational barriers facing parasite-derived therapeutics (41). Continued optimization of nanocarriers, targeted delivery strategies, and controlled-release systems is expected to improve molecular stability, tumor specificity, and therapeutic efficacy, thereby facilitating future clinical application (136) (**Figure 9**).

### 7.4 Manufacturing and regulatory considerations

Beyond biological efficacy, large-scale manufacturing and regulatory approval represent critical prerequisites for the successful clinical translation of parasite-derived therapeutics (53, 54, 111, 135, 140). Owing to their structural complexity and biological origin, PDMs present unique challenges in recombinant production, quality control, standardization, and regulatory evaluation (13, 14). Addressing these issues will be essential to ensure product consistency, safety, and clinical applicability (53, 54, 111). Establishing internationally harmonized manufacturing standards will be critical for future multicenter clinical development (140, 141).

Efficient manufacturing remains one of the primary challenges (13, 53). Many parasite-derived proteins possess complex conformational structures and post-translational modifications that complicate recombinant expression and purification (54, 111). Appropriate selection of expression systems, optimization of culture conditions, and refinement of purification procedures are therefore required to achieve high product yield while preserving biological activity (53, 54). Recombinant expression of parasite-derived immunomodulatory proteins, including cysteine protease inhibitors from *Ascaris lumbricoides*, has demonstrated the feasibility of producing biologically active molecules through optimized recombinant platforms, although further improvements in production efficiency remain necessary (5, 13).

Rigorous quality control is equally important to ensure batch-to-batch consistency and therapeutic safety (111, 142). Standardized evaluation should include assessments of biological activity, purity, endotoxin contamination, structural stability, and long-term storage stability (54, 111). Functional assays confirming the immunomodulatory and antitumor activities of PDMs, together with analytical techniques such as high-performance liquid chromatography, mass spectrometry, and endotoxin testing, are essential components of quality assurance (54, 111). Establishing standardized manufacturing protocols compliant with Good Manufacturing Practice requirements will further facilitate clinical development (54, 141).

Regulatory considerations present additional challenges for parasite-derived therapeutics (117, 118). Because these agents differ substantially from conventional biologics, regulatory agencies such as the U.S. Food and Drug Administration and the European Medicines Agency require comprehensive evidence of product quality, safety, and efficacy before clinical approval (117, 141). Early communication with regulatory authorities, together with well-designed preclinical studies and phased clinical trials, will be important for defining appropriate evaluation criteria and accelerating clinical translation (118, 141). Standardized manufacturing procedures and reproducible quality-control systems will further support regulatory approval while minimizing product variability (111, 140).

Risk management should accompany the entire translational process. Owing to their potent immunomodulatory activities, parasite-derived therapeutics require continuous safety surveillance throughout preclinical and clinical development (3, 13, 123, 124). Comprehensive pharmacovigilance systems should monitor adverse immune reactions, off-target effects, and potential interactions with conventional anticancer therapies (118, 143, 144). In parallel, patient education, long-term follow-up, and multidisciplinary clinical management involving oncologists, immunologists, infectious disease specialists, and parasitologists will facilitate early recognition and management of treatment-related adverse events (5, 138, 139).

Overall, advances in recombinant manufacturing technologies, standardized quality-control systems, and regulatory science will play indispensable roles in facilitating the clinical translation of parasite-derived therapeutics (13, 14, 53, 54, 111). Continued efforts toward scalable production, internationally harmonized regulatory standards, and comprehensive risk-management frameworks are expected to accelerate the safe and successful development of parasite-derived anticancer therapies (117, 118, 138–141).

### 7.5 Future perspectives

Although substantial progress has been made in elucidating the antitumor activities of PDMs, their successful clinical translation will require continued advances in molecular engineering, precision medicine, and interdisciplinary collaboration. Future research should not only focus on improving therapeutic efficacy but also address the challenges associated with safety, patient stratification, and large-scale clinical application (2, 3, 13, 14, 145).

One important direction is the molecular engineering and optimization of PDMs. Advances in protein engineering provide opportunities to improve molecular stability, reduce immunogenicity, and enhance tumor specificity through approaches such as site-directed mutagenesis, fusion protein design, molecular humanization, and the development of bispecific antibody-based therapeutics (13, 146). Conserved parasite-derived proteins such as calreticulin may provide valuable molecular templates for engineering bispecific antibodies capable of simultaneously targeting tumor cells and immune effector cells, thereby expanding future immunotherapeutic strategies (13, 103, 147). Emerging studies also suggest that parasite-derived non-coding RNAs, including microRNAs, represent promising therapeutic candidates for precise regulation of oncogenic signaling pathways (9, 12, 85, 148).

Precision medicine is expected to play an increasingly important role in the clinical application of parasite-derived therapeutics. Comprehensive genomic, transcriptomic, and proteomic analyses may facilitate the identification of tumor subtypes that are most likely to benefit from parasite-derived therapies. The integration of predictive biomarkers, immune profiling, and liquid biopsy technologies could further improve patient stratification, therapeutic monitoring, and individualized treatment optimization (110, 132, 149, 150). These precision medicine approaches may maximize therapeutic efficacy while minimizing unnecessary immune-related adverse effects (3, 13, 14).

Future translational research should also emphasize multidisciplinary collaboration integrating parasitology, immunology, oncology, medicinal chemistry, bioengineering, and computational biology (82, 145). The establishment of standardized PDMs libraries, together with advances in structural biology, bioinformatics, and artificial intelligence-assisted molecular design, may accelerate the discovery and optimization of novel therapeutic candidates (13, 122). Furthermore, multicenter preclinical and clinical studies will be essential to validate therapeutic efficacy, optimize treatment strategies, and establish standardized evaluation systems for future clinical application (13, 82, 145).

Despite encouraging preclinical evidence, clinical studies evaluating PDMs remain extremely limited. Initial phase I studies investigating *Plasmodium*-based immunotherapy in advanced lung cancer and *Toxoplasma gondii*–derived products in melanoma have demonstrated favorable safety and immunogenicity profiles, providing an important proof-of-concept for future clinical development (117, 118, 151–153). As our understanding of host-parasite interactions, tumor immunology, and molecular engineering continues to advance, PDMs are expected to evolve from experimental immunomodulators into rationally engineered therapeutic agents with broad applications in precision cancer immunotherapy (2, 3, 13, 14).

Taken together, the future development of parasite-derived therapeutics will rely on close integration of basic research, bioengineering, translational medicine, and clinical oncology. Continued efforts toward molecular optimization, biomarker-guided precision therapy, standardized clinical evaluation, and interdisciplinary collaboration are expected to accelerate the translation of PDMs into safe and effective anticancer therapies (2, 3, 13, 14, 145).

## 8 Conclusion

PDMs have emerged as a unique and versatile source of bioactive compounds with considerable promise for cancer therapy. As summarized in this review, accumulating evidence demonstrates that parasite-derived proteins, peptides, excretory/secretory products, extracellular vesicles, and other bioactive molecules exert antitumor effects through multiple complementary mechanisms. These include activation of innate and adaptive immune responses, remodeling of the TME, induction of apoptosis and autophagy, inhibition of angiogenesis, suppression of tumor invasion and metastasis, and modulation of tumor metabolic pathways. In summary, these diverse biological activities have been demonstrated across a broad range of preclinical models, including melanoma, lung cancer, CRC, breast cancer, hepatocellular carcinoma, bladder cancer, and hematological malignancies, underscoring the broad translational potential of PDMs.

Current evidence further suggests that the greatest therapeutic value of PDMs may lie in their integration with existing anticancer strategies rather than their use as standalone agents. Their capacity to enhance antitumor immunity, alleviate immunosuppressive TMEs, and improve responses to immune checkpoint inhibitors, chemotherapy, and other targeted therapies provides a strong rationale for combination-based therapeutic approaches. Moreover, recent advances in protein engineering, nanotechnology-based delivery systems, and synthetic biology offer new opportunities to improve molecular stability, tumor specificity, and pharmacokinetic performance, thereby facilitating future clinical translation.

Despite these encouraging advances, several important challenges remain before PDMs can be translated into routine clinical practice. Future studies should further elucidate their molecular mechanisms of action, optimize delivery strategies, minimize potential immunogenicity and off-target effects, establish standardized manufacturing and quality-control procedures, and comprehensively evaluate long-term safety and efficacy in well-designed preclinical and clinical studies. In parallel, the identification of predictive biomarkers and incorporation of precision medicine strategies will be essential for selecting patient populations most likely to benefit from parasite-derived therapies.

Overall, PDMs represent a rapidly expanding class of biologically active compounds that provide new opportunities for cancer immunotherapy and translational oncology. Continued interdisciplinary collaboration among parasitology, immunology, oncology, bioengineering, and pharmaceutical sciences will be essential to accelerate their development and clinical application. Although substantial challenges remain, continued mechanistic investigation together with rigorous translational research is expected to facilitate the development of safe, effective, and clinically applicable parasite-derived therapeutics that complement existing treatment modalities and broaden future therapeutic options for patients with cancer.

## References

[1] Tufail M Jiang CH Li N . Immune evasion in cancer: mechanisms and cutting-edge therapeutic approaches. Signal Transduct Target Ther. (2025) 10:227. doi: 10.1038/s41392-025-02280-1 40739089 PMC12311175

[2] Eissa MM El-Faham MH El Skhawy N . Bridging the gap for diverse applications of parasites as advanced cancer therapeutics: current progress and future directions. Infect Agent Cancer. (2025) 20:53. doi: 10.1186/s13027-025-00679-7 40731364 PMC12309174

[3] Pawłowska M Jarek D Milanowski J Szewczyk-Golec K . Parasitic infections and carcinogenesis: molecular mechanisms, immune modulation, and emerging therapeutic strategies. Oncol Res. (2026) 34:8. doi: 10.32604/or.2025.071891 41613796 PMC12848758

[4] Ding H Wu S Jin Z Zheng B Hu Y He K . Anti-tumor effect of parasitic protozoans. Bioengineering (Basel). (2022) 9:395. doi: 10.3390/bioengineering9080395 36004920 PMC9405343

[5] Sharma M Khurana S . Immunomodulation by helminthic parasites and worm therapy. Trop Parasitol. (2025) 15:2–7. doi: 10.4103/tp.tp_5_25 40433639 PMC12105771

[6] Douglas B Oyesola O Cooper MM Posey A Tait Wojno E Giacomin PR . Immune system investigation using parasitic helminths. Annu Rev Immunol. (2021) 39:639–65. doi: 10.1146/annurev-immunol-093019-122827 33646858 PMC8162934

[7] Joardar N Mondal C Sinha Babu SP . A review on the interactions between dendritic cells, filarial parasite and parasite-derived molecules in regulating the host immune responses. Scand J Immunol. (2021) 93:e13001. doi: 10.1111/sji.13001 33247468

[8] Mu Y McManus DP Hou N Cai P . Schistosome infection and schistosome-derived products as modulators for the prevention and alleviation of immunological disorders. Front Immunol. (2021) 12:619776. doi: 10.3389/fimmu.2021.619776 33692793 PMC7937812

[9] Hu C Li Y Pan D Wang J Zhu L Lin Y . A Schistosoma japonicum microRNA exerts antitumor effects through inhibition of both cell migration and angiogenesis by targeting PGAM1. Front Oncol. (2021) 11:652395. doi: 10.3389/fonc.2021.652395 34221971 PMC8242254

[10] Wu H Li M Shao X An Z Du J Yin H . Trichinella spiralis muscle larvae excretory/secretory products trigger apoptosis and S-phase arrest of the non-small-cell lung cancer line A549. Exp Parasitol. (2020) 218:107983. doi: 10.1016/j.exppara.2020.107983 32861680

[11] Ding J Tang B Liu X Bai X Wang Y Li S . Excretory-secretory product of Trichinella spiralis inhibits tumor cell growth by regulating the immune response and inducing apoptosis. Acta Trop. (2022) 225:106172. doi: 10.1016/j.actatropica.2021.106172 34627760

[12] Jiang P Wang J Zhu S Hu C Lin Y Pan W . Identification of a Schistosoma japonicum microRNA that suppresses hepatoma cell growth and migration by targeting host FZD4 gene. Front Cell Infect Microbiol. (2022) 12:786543. doi: 10.3389/fcimb.2022.786543 35174106 PMC8842725

[13] Li J El Shanawany EE Hassan SE Li PY Sun JH Li HM . Toxoplasma gondii as a drug for anti-tumor immunotherapy: mechanisms, challenges, and perspectives. Parasite. (2026) 33:4. doi: 10.1051/parasite/2026006 41641877 PMC12875063

[14] Xie Y Wang J Wang Y Wen Y Pu Y Wang B . Parasite-enhanced immunotherapy: transforming the “cold” tumors to “hot” battlefields. Cell Commun Signal. (2024) 22:448. doi: 10.1186/s12964-024-01822-4 39327550 PMC11426008

[15] Lu X Zhang Z Zhang Y Wang L Hu Y Wang Y . Antitumor effects of leishmania donovani by inducing a pro-inflammatory tumor microenvironment in hepatocellular carcinoma. Acta Trop. (2026) 279:108125. doi: 10.1016/j.actatropica.2026.108125 42119960

[16] Zhang J Zheng W Gong Y Jia X Liu H Guan F . A hidden “promoter”: Schistosoma japonicum soluble egg antigen activates MAPK/PI3K-AKT pathways and inhibits autophagy to facilitate colorectal cancer. Infect Immun. (2026) 94:e0069625. doi: 10.1128/iai.00696-25 41769997 PMC13081734

[17] Kim JS Lee D Kim D Mun SJ Cho E Son W . Toxoplasma gondii GRA8-derived peptide immunotherapy improves tumor targeting of colorectal cancer. Oncotarget. (2020) 11:62–73. doi: 10.18632/oncotarget.27417 32002124 PMC6967779

[18] Wang B Li Q Wang J Zhao S Nashun B Qin L . Plasmodium infection inhibits tumor angiogenesis through effects on tumor-associated macrophages in a murine implanted hepatoma model. Cell Commun Signal. (2020) 18:157. doi: 10.1186/s12964-020-00570-5 32972437 PMC7513281

[19] Qian W Chen Y Li C Li X Lv C Jia Y . Neospora caninum inhibits Lewis cancer and B16f10 melanoma lung metastasis development by activating the immune response in murine models. Acta Parasitol. (2025) 70:52. doi: 10.1007/s11686-025-00996-2 39918646

[20] Chen M Yang P Xin Z Chen J Zou W Zhou L . Toxoplasma gondii gra5 deletion mutant protects hosts against Toxoplasma gondii infection and breast tumors. Front Immunol. (2023) 14:1173379. doi: 10.3389/fimmu.2023.1173379 37426671 PMC10327641

[21] Tung CL Chao WY Li YZ Shen CH Zhao PW Chen SH . Ivermectin induces cell cycle arrest and caspase-dependent apoptosis in human urothelial carcinoma cells. Int J Med Sci. (2022) 19:1567–75. doi: 10.7150/ijms.76623 36185334 PMC9515697

[22] Payne SN Emmerich PB Davis NM Deming DA Knoll LJ . Novel murine pancreatic tumor model demonstrates immunotherapeutic control of tumor progression by a Toxoplasma gondii protein. Infect Immun. (2021) 89:e0050821. doi: 10.1128/IAI.00508-21 34543124 PMC8594601

[23] Rchiad Z Haidar M Ansari HR Tajeri S Mfarrej S Ben Rached F . Novel tumour suppressor roles for GZMA and RASGRP1 in Theileria annulata-transformed macrophages and human B lymphoma cells. Cell Microbiol. (2020) 22:e13255. doi: 10.1111/cmi.13255 32830401 PMC7685166

[24] Sun HC Deng PM Fu Y Deng JH Xie RH Huang J . Protective efficacy of Toxoplasma gondii GRA12 or GRA7 recombinant proteins encapsulated in PLGA nanoparticles against acute Toxoplasma gondii infection in mice. Front Cell Infect Microbiol. (2023) 13:1209755. doi: 10.3389/fcimb.2023.1209755 37502604 PMC10368986

[25] Kong L Jiang D He C Xia J Wei H Zhou L . TgROP18 targets IL20RB for host-defense-related-STAT3 activation during Toxoplasma gondii infection. Parasit Vectors. (2020) 13:400. doi: 10.1186/s13071-020-04251-7 32767999 PMC7412674

[26] Hasan T Shimoda N Nakamura S Fox BA Bzik DJ Ushio-Watanabe N . Protective efficacy of recombinant Toxoplasma gondii dense granule protein 15 against toxoplasmosis in C57BL/6 mice. Vaccine. (2024) 42:2299–309. doi: 10.1016/j.vaccine.2024.02.062 38429153

[27] Jhandi S Vera BA Galindo J Montoya JG Contopoulos-Ioannidis DG . Toxoplasma gondii and a cancer biology dichotomy: a systematic review of experimental studies of its antitumor and pro-tumor effects. Pathogens. (2026) 15:351. doi: 10.3390/pathogens15040351 42075678 PMC13118916

[28] Eissa MM Gaafar MR Younis LK Ismail CA El Skhawy N . Prophylactic antineoplastic activity of Toxoplasma gondii RH derived antigen against ehrlich solid carcinoma with evidence of shared antigens by comparative immunoblotting. Infect Agent Cancer. (2023) 18:21. doi: 10.1186/s13027-023-00500-3 37029378 PMC10082516

[29] Xu LQ Yao LJ Jiang D Zhou LJ Chen M Liao WZ . A uracil auxotroph Toxoplasma gondii exerting immunomodulation to inhibit breast cancer growth and metastasis. Parasit Vectors. (2021) 14:601. doi: 10.1186/s13071-021-05032-6 34895326 PMC8665513

[30] Antonelli LR Junqueira C Vinetz JM Golenbock DT Ferreira MU Gazzinelli RT . The immunology of Plasmodium vivax malaria. Immunol Rev. (2020) 293:163–89. doi: 10.1111/imr.12816 31642531

[31] Cordery DV Urban BC . Immune recognition of Plasmodium-infected erythrocytes. Adv Exp Med Biol. (2009) 653:175–84. doi: 10.1007/978-1-4419-0901-5_12 19799119

[32] Wu R Chen X Chen H Li M Liang Y . Plasmodium infection downregulates hypoxia–inducible factor 1α expression to suppress the vascularization and tumorigenesis of liver cancer. Oncol Lett. (2024) 28:604. doi: 10.3892/ol.2024.14737 39483968 PMC11525613

[33] Rios-Barros LV Chiaradia RM Serravite AM Horta MF Castro-Gomes T . Leishmania plasma membrane - general composition, structure and biological functions. Curr Top Membr. (2025) 95:215–47. doi: 10.1016/bs.ctm.2025.06.009 40774761

[34] Matte C Arango Duque G Descoteaux A . Leishmania donovani metacyclic promastigotes impair phagosome properties in inflammatory monocytes. Infect Immun. (2021) 89:e0000921. doi: 10.1128/IAI.00009-21 33875473 PMC8208509

[35] Silveira MB Gomes RS Shio MT Rugani JN Paranaiba LF Soares RP . Lipophosphoglycan from dermotropic New World Leishmania upregulates interleukin-32 and proinflammatory cytokines through TLR4 and NOD2 receptors. Front Cell Infect Microbiol. (2022) 12:805720. doi: 10.3389/fcimb.2022.805720 35402314 PMC8983857

[36] Devsani N Vemula D Bhandari V . The glycoprotein gp63- a potential pan drug target for developing new antileishmanial agents. Biochimie. (2023) 207:75–82. doi: 10.1016/j.biochi.2022.11.015 36473603

[37] Antonia AL Barnes AB Martin AT Wang L Ko DC . Variation in Leishmania chemokine suppression driven by diversification of the GP63 virulence factor. PloS NeglTrop Dis. (2021) 15:e0009224. doi: 10.1371/journal.pntd.0009224 34710089 PMC8577781

[38] Soto-Serna LE Diupotex M Zamora-Chimal J Ruiz-Remigio A Delgado-Domínguez J Cervantes-Sarabia RB . Leishmania mexicana: novel insights of immune modulation through amastigote exosomes. J Immunol Res. (2020), 8894549. doi: 10.1155/2020/8894549 33344659 PMC7728480

[39] Emerson LE Gioseffi A Barker H Sheppe A Morrill JK Edelmann MJ . Leishmania infection-derived extracellular vesicles drive transcription of genes involved in M2 polarization. Front Cell Infect Microbiol. (2022) 12:934611. doi: 10.3389/fcimb.2022.934611 36093197 PMC9455154

[40] Pereira-Silva GC Medina JM Paschoaletto L Mangeth L Coelho FS Attias M . Leishmania amazonensis-derived extracellular vesicles (EVs) induce neutrophil extracellular traps (NETs). J Leukoc Biol. (2024) 117:qiae196. doi: 10.1093/jleuko/qiae196 39241110

[41] Greening DW Xu R Ale A Hagemeyer CE Chen W . Extracellular vesicles as next generation immunotherapeutics. Semin Cancer Biol. (2023) 90:73–100. doi: 10.1016/j.semcancer.2023.02.002 36773820

[42] Caner A Sadqova A Erdoan A Namlses D Nalbantsoy A Oltulu F . Targeting of antitumor immune responses with live-attenuated Leishmania strains in breast cancer model. Breast Cancer. (2020) 27:1082–95. doi: 10.1007/s12282-020-01112-0 32472473

[43] Skelly PJ Da’dara AA . Schistosome secretomes. Acta Trop. (2022) 236:106676. doi: 10.1016/j.actatropica.2022.106676 36113567

[44] White MPJ McManus CM Maizels RM . Regulatory T-cells in helminth infection: induction, function and therapeutic potential. Immunology. (2020) 160:248–60. doi: 10.1111/imm.13190 32153025 PMC7341546

[45] Pekkle Lam HY Liang TR Jiang SJ Peng SY . Schistosoma mansoni soluble egg antigen suppresses colorectal cancer growth *in vitro* and *in vivo*. J Microbiol Immunol Infect. (2025) 58:241–50. doi: 10.1016/j.jmii.2024.11.009 39653602

[46] Ge P Ong CY Abdalkareem AE Khoo BY Yuan B . IFN-γ and IL-18 in conditioned media of parasite-infected host and IL-21-silenced colorectal cancer cells. Exp Ther Med. (2021) 21:103. doi: 10.3892/etm.2020.9535 33335566 PMC7739864

[47] Mendoza-Rodríguez MG Medina-Reyes D Sánchez-Barrera CA Fernández-Muñoz KV García-Castillo V Ledesma-Torres JL . Helminth-derived molecules improve 5-fluorouracil treatment on experimental colon tumorigenesis. BioMed Pharmacother. (2024) 175:116628. doi: 10.1016/j.biopha.2024.116628 38663106

[48] Yue T Wang J Liu F Gong P Li J Zhang X . The effects of anti-lung cancer in nude mice by a fully human single-chain antibody against associated antigen Ts7TMR between A549 cells and Trichinella spiralis. Artif Cells Nanomed Biotechnol. (2024) 52:300–8. doi: 10.1080/21691401.2024.2347377 38753524

[49] Ajam Z Tavakoli-Yaraki M Razmjou E Hosseini ES Alipour M Meamar AR . Assessing the impact of Fasciola hepatica excretory-secretory antigens on A549 cells: viability, apoptosis, ROS, and stem cell markers. BMC Res Notes. (2026) 19:281. doi: 10.1186/s13104-026-07872-w 42129907 PMC13339426

[50] Carmona C Dowd AJ Smith AM Dalton JP . Cathepsin L proteinase secreted by Fasciola hepatica *in vitro* prevents antibody-mediated eosinophil attachment to newly excysted juveniles. Mol Biochem Parasitol. (1993) 62:9–17. doi: 10.1016/0166-6851(93)90172-t 8114830

[51] Dalton JP O'Neill S Stack C Collins P Walshe A Sekiya M . Fasciola hepatica cathepsin L-like proteases: biology, function, and potential in the development of first generation liver fluke vaccines. Int J Parasitol. (2003) 33:1173–81. doi: 10.1016/s0020-7519(03)00171-1 13678633

[52] Ab Talib NN Nisha M Ramasamy R Pang JC . Preliminary studies on the effect of excretory secretory (ES) Ascaris lumbricoides antigens on colorectal cell line viability. Trop BioMed. (2024) 41:160–5. doi: 10.47665/tb.41.2.005 39154268

[53] Ahumada V Zakzuk J Aglas L Coronado S Briza P Regino R . Comparison of antibody responses against two molecules from Ascaris lumbricoides: The allergen Asc l 5 and the immunomodulatory protein Al-CPI. Biol (Basel). (2023) 12:1340. doi: 10.3390/biology12101340 37887050 PMC10604738

[54] Cortes-Selva D Fairfax K . Schistosome and intestinal helminth modulation of macrophage immunometabolism. Immunology. (2021) 162:123–34. doi: 10.1111/imm.13231 32614982 PMC7808165

[55] Ranasinghe SL McManus DP . Echinococcus granulosus: Cure for cancer revisited. Front Med (Lausanne). (2018) 5:60. doi: 10.3389/fmed.2018.00060 29594121 PMC5857532

[56] Guan W Zhang X Wang X Lu S Yin J Zhang J . Employing parasite against cancer: A lesson from the canine tapeworm Echinococcus granulocus. Front Pharmacol. (2019) 10:1137. doi: 10.3389/fphar.2019.01137 31607934 PMC6774290

[57] Rashno Z Rismani E Ghasemi JB Mansouri M Shabani M Afgar A . Design of ion channel blocking, toxin-like Kunitz inhibitor peptides from the tapeworm, Echinococcus granulosus, with potential anti-cancer activity. Sci Rep. (2023) 13:11465. doi: 10.1038/s41598-023-38159-w 37454225 PMC10349847

[58] Mosajakhah H Shanehbandi D Ahmadpour E Mahami-Oskouei M Sadeghi K Spotin A . MicroRNA-145 enhances lung cancer cell progression after exposure to lyophilized fertile hydatid cyst fluid of Echinococcus granulosus sensu stricto. Exp Parasitol. (2024) 265:108829. doi: 10.1016/j.exppara.2024.108829 39179144

[59] Ghosh SK Shukla D Mahor H Srivastava SK Bodhale N Banerjee R . Leishmania surface molecule lipophosphoglycan-TLR2 interaction moderates TPL2-mediated TLR2 signalling for parasite survival. Immunology. (2024) 171:117–30. doi: 10.1111/imm.13702 37849037

[60] Virnik K Zhou W Medvedev A Walsh G Perry-Anderson J Majam V . Live attenuated rubella vectors expressing Plasmodium falciparum circumsporozoite protein (Pf-CSP) provide a novel malaria vaccine platform in the rhesus macaque. Biochem Biophys Res Commun. (2021) 577:58–63. doi: 10.1016/j.bbrc.2021.08.052 34507066 PMC10167915

[61] Żelechowska P Góralczyk-Bińkowska A . Mast cell response to parasites: From recognition and activation to host defense modulation. Cell Physiol Biochem. (2025) 59:631–51. doi: 10.33594/000000815 41047922

[62] Camaya I Mok TY Lund M To J Braidy N Robinson MW . The parasite-derived peptide FhHDM-1 activates the PI3K/Akt pathway to prevent cytokine-induced apoptosis of β-cells. J Mol Med (Berl). (2021) 99:1605–21. doi: 10.1007/s00109-021-02122-x 34374810

[63] Liu X Jiang Y Ye J Wang X . Helminth infection and helminth-derived products: A novel therapeutic option for non-alcoholic fatty liver disease. Front Immunol. (2022) 13:999412. doi: 10.3389/fimmu.2022.999412 36263053 PMC9573989

[64] Bunte MJM Schots A Kammenga JE Wilbers RHP . Helminth glycans at the host-parasite interface and their potential for developing novel therapeutics. Front Mol Biosci. (2021) 8:807821. doi: 10.3389/fmolb.2021.807821 35083280 PMC8784694

[65] Wu J Zhu Y Zhou L Lu Y Feng T Dai M . Parasite-derived excretory-secretory products alleviate gut microbiota dysbiosis and improve cognitive impairment induced by a high-fat diet. Front Immunol. (2021) 12:710513. doi: 10.3389/fimmu.2021.710513 34745091 PMC8564115

[66] Ledesma-Soto Y Chávez-Soto I Calderón-Torres M Rodríguez-Lozoya AM Olguin JE Hernández-Portilla LB . Intact glycoconjugates from Taenia crassiceps excreted/secreted products ameliorate chemically induced colitis by modulating inflammation and strengthening adherens junctions. Inflammopharmacology. (2025) 33:4725–47. doi: 10.1007/s10787-025-01821-y 40576745 PMC12397184

[67] Goto Y . Immunomodulation by Leishmania parasites: Potential for controlling other diseases. Parasitol Int. (2025) 104:102987. doi: 10.1016/j.parint.2024.102987 39515578

[68] Steinfelder S Andersen JF Cannons JL Feng CG Joshi M Dwyer D . The major component in schistosome eggs responsible for conditioning dendritic cells for Th2 polarization is a T2 ribonuclease (omega-1). J Exp Med. (2009) 206:1681–90. doi: 10.1084/jem.20082462 19635859 PMC2722182

[69] Everts B Hussaarts L Driessen NN Meevissen MH Schramm G van der Ham AJ . Schistosome-derived omega-1 drives Th2 polarization by suppressing protein synthesis following internalization by the mannose receptor. J Exp Med. (2012) 209:1753–S1. doi: 10.1084/jem.20111381 22966004 PMC3457738

[70] Soto-Olguín N Zamora-Chimal J Delgado-Domínguez J Becker I . Leishmania mexicana lipophosphoglycan activates dermal γδ T cells with participation of TLR2. Acta Parasitol. (2023) 68:122–9. doi: 10.1007/s11686-022-00639-w 36434381

[71] Bhattacharya P Gannavaram S Ismail N Saxena A Dagur PK Akue A . Toll-like receptor-9 (TLR-9) signaling is crucial for inducing protective immunity following immunization with genetically modified live attenuated Leishmania parasites. Pathogens. (2023) 12:534. doi: 10.3390/pathogens12040534 37111420 PMC10143410

[72] Teufel LU Joosten LAB Dos Santos JC . Differential structure and immunomodulatory functions of lipophosphoglycan between Leishmania spp. Immunol Lett. (2024) 268:106885. doi: 10.1016/j.imlet.2024.106885 38901739

[73] Rêgo FD Cardoso CDA Moreira POL Nogueira PM Araújo MS Borges VM . Leishmania amazonensis from distinct clinical forms/hosts has polymorphisms in lipophosphoglycans, displays variations in immunomodulatory properties and, susceptibility to antileishmanial drugs. Cell Biol Int. (2022) 46:1947–58. doi: 10.1002/cbin.11880 35998255 PMC9804363

[74] Pan J Ma M Qin L Kang Z Adah D Tao Z . Plasmodium infection inhibits triple negative 4T1 breast cancer potentially through induction of CD8+ T cell-mediated antitumor responses in mice. BioMed Pharmacother. (2021) 138:111406. doi: 10.1016/j.biopha.2021.111406 33676307

[75] Li K Feng X Hikosaka K Norose K . Murine model of primary acquired ocular toxoplasmosis: Fluorescein angiography and multiplex immune mediator profiles in the aqueous humor. Invest Ophthalmol Vis Sci. (2021) 62:9. doi: 10.1167/iovs.62.3.9 33683297 PMC7960860

[76] Ashkenazi-Preiser H Mikula I Baniyash M . The diverse roles of myeloid derived suppressor cells in mucosal immunity. Cell Immunol. (2021) 365:104361. doi: 10.1016/j.cellimm.2021.104361 33984533

[77] Wang H Zhu Y Li M Pan J Li D Guo WP . Transcriptome profiling of A549 non-small cell lung cancer cells in response to Trichinella spiralis muscle larvae excretory/secretory products. Front Vet Sci. (2023) 10:1208538. doi: 10.3389/fvets.2023.1208538 37601754 PMC10433203

[78] Ruenchit P Reamtong O Khowawisetsut L Adisakwattana P Chulanetra M Kulkeaw K . Peptide of Trichinella spiralis infective larval extract that harnesses growth of human hepatoma cells. Front Cell Infect Microbiol. (2022) 12:882608. doi: 10.3389/fcimb.2022.882608 35558100 PMC9086976

[79] Liu Z Zhang L Liang Y Lu L . Pathology and molecular mechanisms of Schistosoma japonicum-associated liver fibrosis. Front Cell Infect Microbiol. (2022) 12:1035765. doi: 10.3389/fcimb.2022.1035765 36389166 PMC9650140

[80] Peterková K Konečný L Macháček T Jedličková L Winkelmann F Sombetzki M . Winners vs. losers: Schistosoma mansoni intestinal and liver eggs exhibit striking differences in gene expression and immunogenicity. PloS Pathog. (2024) 20:e1012268. doi: 10.1371/journal.ppat.1012268 38814989 PMC11166329

[81] Jain S . Is Schistosoma mansoni playing a part in liver carcinogenesis? J Helminthol. (2024) 98:e61. doi: 10.1017/S0022149X24000506 39469749

[82] Yan L Li Y Yang X Li R Zhu C He X . PI3K/AKT signaling in parasites and parasite diseases: Role and therapeutic potential. Virulence. (2025) 16:2532803. doi: 10.1080/21505594.2025.2532803 40643963 PMC12269711

[83] Doherty CM Patterson PR Emeanuwa JA Belmares Ortega J Fox BA Bzik DJ . T lymphocyte-dependent IL-10 down-regulates a cytokine storm driven by Toxoplasma gondii GRA24. mBio. (2024) 15:e0145524. doi: 10.1128/mbio.01455-24 39440975 PMC11559025

[84] Urdapilleta AAA Santos Alfani AO Barroso DH Vinecky F Amaral Vaz Bandeira SDG Andrade AC . Treatment of refractory mucosal leishmaniasis is associated with parasite overexpression of HSP70 and ATPase and reduced host hydrogen peroxide production (Brief Report). Biomedicines. (2024) 12:2227. doi: 10.3390/biomedicines12102227 39457540 PMC11504370

[85] Zhong H Dong B Zhu D Fu Z Liu J Guan G . Schistosoma japonicumsja-let-7 inhibits the growth of hepatocellular carcinoma cells via cross-species regulation of Col1α2. Genes (Basel). (2024) 15:1165. doi: 10.3390/genes15091165 39336756 PMC11431810

[86] Vega-Benedetti AF Loi E Zavattari P . DNA methylation alterations caused by Leishmania infection may generate a microenvironment prone to tumour development. Front Cell Infect Microbiol. (2022) 12:984134. doi: 10.3389/fcimb.2022.984134 36105147 PMC9465093

[87] Mercado-Camargo J Cervantes-Ceballos L Vivas-Reyes R Pedretti A Serrano-García ML Gómez-Estrada H . Homology modeling of leishmanolysin (gp63) from Leishmania panamensis and molecular docking of flavonoids. ACS Omega. (2020) 5:14741–9. doi: 10.1021/acsomega.0c01584 32596611 PMC7315592

[88] Anton L Cobb DW Ho CM . Structural parasitology of the malaria parasite Plasmodium falciparum. Trends Biochem Sci. (2022) 47:149–59. doi: 10.1016/j.tibs.2021.10.006 34887149 PMC11236216

[89] Neveu G Lavazec C . Erythroid cells and malaria parasites: It’s a match! Curr Opin Hematol. (2021) 28:158–63. doi: 10.1097/MOH.0000000000000641 33631784

[90] Martínez-Flórez A Galizzi M Izquierdo L Bustamante JM Rodriguez A Rodriguez F . Repurposing bioenergetic modulators against protozoan parasites responsible for tropical diseases. Int J Parasitol Drugs Drug Resist. 14:17–27. doi: 10.1016/j.ijpddr.2020.07.002 32829099 PMC7452664

[91] Lima TS Mallya S Jankeel A Messaoudi I Lodoen MB . Toxoplasma gondii extends the life span of infected human neutrophils by inducing cytosolic PCNA and blocking activation of apoptotic caspases. mBio. (2021) 12:e02031–20. doi: 10.1128/mBio.02031-20 33500339 PMC7858050

[92] Rashidi S Mansouri R Ali-Hassanzadeh M Mojtahedi Z Shafiei R Savardashtaki A . The host mTOR pathway and parasitic diseases pathogenesis. Parasitol Res. (2021) 120:1151–66. doi: 10.1007/s00436-021-07070-6 33534053 PMC7856335

[93] Yang X Ding W Qian X Jiang P Chen Q Zhang X . Schistosoma japonicum infection leads to the reprogramming of glucose and lipid metabolism in the colon of mice. Front Vet Sci. (2021) 8:645807. doi: 10.3389/fvets.2021.645807 33791356 PMC8006365

[94] Silvestre A Shintre SS Rachidi N . Released parasite-derived kinases as novel targets for antiparasitic therapies. Front Cell Infect Microbiol. (2022) 12:825458. doi: 10.3389/fcimb.2022.825458 35252034 PMC8893276

[95] Zaichikova SG Donenko FV Kormosh NG Kiselevskii MV . Effect of cathepsine L1 on transformation of Malignant melanoma B16 cells into melanocytes. Bull Exp Biol Med. (2020) 169:254–7. doi: 10.1007/s10517-020-04862-1 32651814

[96] Li Y Zhang Y Xia N Zhou T Shen B . Antitumor effects of a Toxoplasma mutant lacking lactate dehydrogenases. Parasitol Res. (2021) 120:3335–9. doi: 10.1007/s00436-021-07283-9 34405281

[97] Eissa MM Allam SRA Ismail CA Ghazala RA El Skhawy N Ibrahim EIE . Molecular mimicry between parasites and cancer: A novel approach for developing cancer vaccines and therapeutic antibodies. Cancer Immunol Immunother. (2025) 74:212. doi: 10.1007/s00262-025-04069-1 40402283 PMC12098237

[98] Jacobs BA Prince S Smith KA . Gastrointestinal nematode-derived antigens alter colorectal cancer cell proliferation and migration through regulation of cell cycle and epithelial-mesenchymal transition proteins. Int J Mol Sci. (2020) 21:7845. doi: 10.3390/ijms21217845 33105843 PMC7660063

[99] Doleschel D Hoff S Koletnik S Rix A Zopf D Kiessling F . Regorafenib enhances anti-PD1 immunotherapy efficacy in murine colorectal cancers and their combination prevents tumor regrowth. J Exp Clin Cancer Res. (2021) 40:288. doi: 10.1186/s13046-021-02043-0 34517894 PMC8436536

[100] Yu Q Tang R Mo W Zhao L Li L . Baicalein enhances radiosensitivity in colorectal cancer via JAK2/STAT3 pathway inhibition. Chem Biol Drug Des. (2024) 104:e14611. doi: 10.1111/cbdd.14611 39152534

[101] Ma X Zhou Z Zhang X Fan M Hong Y Feng Y . Sodium butyrate modulates gut microbiota and immune response in colorectal cancer liver metastatic mice. Cell Biol Toxicol. (2020) 36:509–15. doi: 10.1007/s10565-020-09518-4 32172331

[102] Chellan P Avery VM Duffy S Land KM Tam CC Kim JH . Bioactive half-sandwich Rh and Ir bipyridyl complexes containing artemisinin. J Inorg Biochem. (2021) 219:111408. doi: 10.1016/j.jinorgbio.2021.111408 33826972

[103] Schcolnik-Cabrera A Juárez M Oldak B Cruz-Rivera M Flisser A Dueñas-González A . *In vitro* employment of recombinant Taenia solium calreticulin as a novel strategy against breast and ovarian cancer stem-like cells. Arch Med Res. (2020) 51:65–75. doi: 10.1016/j.arcmed.2019.12.003 32097797

[104] Crotts MS Jacobs JC Baer RW Cox JL . Doramectin induces apoptosis in B16 melanoma cells. Anti-Cancer Agents Med Chem. (2025) 25:244–56. doi: 10.2174/0118715206325844240909144543 39411968

[105] Nordmaj MA Roberts ME Sachse ES Dagil R Andersen AP Skeltved N . Development of a bispecific immune engager using a recombinant malaria protein. Cell Death Dis. (2021) 12:353. doi: 10.1038/s41419-021-03611-0 33824272 PMC8024270

[106] Yu Q Chen S Yu L Xu D Yin S . Cathepsin L-activatable fluorescent probe for real-time monitoring of drug-resistant breast cancer cells. Chemistry. (2025) 31:e202501624. doi: 10.1002/chem.202501624 40627318

[107] Wang C Hong Y Dong L Cheng H Jin D Zhao R . An AND-gate bioluminescent probe for precise tumor imaging. Chem Sci. (2023) 14:5768–73. doi: 10.1039/d3sc00556a 37265734 PMC10231332

[108] Suarez-Martinez AD Sole-Gras M Dykes SS Wakefield ZR Bauer K Majbour D . Bioprinting on live tissue for investigating cancer cell dynamics. Tissue Eng Part A. (2021) 27:438–53. doi: 10.1089/ten.TEA.2020.0190 33059528 PMC8349716

[109] Weingartner M Stücheli S Jebbawi F Gottstein B Beldi G Lundström-Stadelmann B . Albendazole reduces hepatic inflammation and endoplasmic reticulum-stress in a mouse model of chronic Echinococcus multilocularis infection. PloS NeglTrop Dis. (2022) 16:e0009192. doi: 10.1371/journal.pntd.0009192 35030165 PMC8794265

[110] Bruno PS Biggers P Nuru N Versaci N Chirila MI Darie CC . Small biological fighters against cancer: Viruses, bacteria, archaea, fungi, protozoa, and microalgae. Biomedicines. (2025) 13:665. doi: 10.3390/biomedicines13030665 40149641 PMC11940145

[111] Myhill LJ Jensen P Zakeri A Nielsen LF Jakobsen SR Mejer H . Effects of the dietary fibre inulin and Trichuris suis products on inflammatory responses in lipopolysaccharide-stimulated macrophages. Mol Immunol. (2020) 121:127–35. doi: 10.1016/j.molimm.2020.03.006 32200170

[112] Yaron JR Zhang L Guo Q Burgin M Schutz LN Awo E . Deriving immune modulating drugs from viruses-a new class of biologics. J Clin Med. (2020) 9:972. doi: 10.3390/jcm9040972 32244484 PMC7230489

[113] Wellenberg A Weides L Kurzke J Hennecke T Bornhorst J Crone B . Use of C. elegans as a 3R-compliant *in vivo* model for the chemoprevention of cisplatin-induced neurotoxicity. Exp Neurol. (2021) 341:113705. doi: 10.1016/j.expneurol.2021.113705 33753139

[114] Jebbawi F Bellanger AP Lunström-Stadelmann B Rufener R Dosch M Goepfert C . Innate and adaptive immune responses following PD-L1 blockade in treating chronic murine alveolar echinococcosis. Parasite Immunol. (2021) 43:e12834. doi: 10.1111/pim.12834 33754355

[115] Jubel JM Barbati ZR Burger C Wirtz DC Schildberg FA . The role of PD-1 in acute and chronic infection. Front Immunol. (2020) 11:487. doi: 10.3389/fimmu.2020.00487 32265932 PMC7105608

[116] da Fonseca-Martins AM Ramos TD Pratti JES Firmino-Cruz L Gomes DCO Soong L . Immunotherapy using anti-PD-1 and anti-PD-L1 in Leishmania amazonensis-infected BALB/c mice reduce parasite load. Sci Rep. (2019) 9:20275. doi: 10.1038/s41598-019-56336-8 31889072 PMC6937231

[117] Kauffman KD Sakai S Lora NE Namasivayam S Baker PJ Kamenyeva O . PD-1 blockade exacerbates Mycobacterium tuberculosis infection in rhesus macaques. Sci Immunol. (2021) 6:eabf3861. doi: 10.1126/sciimmunol.abf3861 33452107 PMC8300572

[118] Anidi IU Sakai S Brooks K Fling SP Wagner MJ Lurain K . Exacerbation of CMV and nontuberculous mycobacterial infections following PD-1 blockade for HIV-associated Kaposi sarcoma. Open Forum Infect Dis. (2024) 11:ofae183. doi: 10.1093/ofid/ofae183 38680611 PMC11049581

[119] Mahasongkram K Glab-Ampai K Kaewchim K Saenlom T Chulanetra M Sookrung N . Agonistic bivalent human scFvs-Fcγ fusion antibodies to OX40 ectodomain enhance T cell activities against cancer. Vaccines (Basel). (2023) 11:1826. doi: 10.3390/vaccines11121826 38140230 PMC10747724

[120] Chen S Liu L Zhang W Sun L Wang F Zhao Y . Suppressed dendritic cell functions by cystatin C lead to compromised immunity *in vivo*. Cell Immunol. (2020) 349:104049. doi: 10.1016/j.cellimm.2020.104049 32057353

[121] Wan S Sun X Tang W Wang L Wu Z Sun X . Exosome-depleted excretory-secretory products of the fourth-stage larval Angiostrongylus cantonensis promotes alternative activation of macrophages through metabolic reprogramming by the PI3K-Akt pathway. Front Immunol. (2021) 12:685984. doi: 10.3389/fimmu.2021.685984 34367145 PMC8343011

[122] Fernandes RS de Assis Burle-Caldas G Sergio SAR Bráz AF da Silva Leite NP Pereira M . The immunogenic potential of an optimized mRNA lipid nanoparticle formulation carrying sequences from virus and protozoan antigens. J Nanobiotechnol. (2025) 23:221. doi: 10.1186/s12951-025-03201-8 40102899 PMC11921523

[123] Motta RV Culver EL . IgG4 autoantibodies and autoantigens in the context of IgG4-autoimmune disease and IgG4-related disease. Front Immunol. (2024) 15:1272084. doi: 10.3389/fimmu.2024.1272084 38433835 PMC10904653

[124] Philip CM Eapen M S S . Bilious mask: parasite masquerading as Malignant gall bladder polyp. BMJ Case Rep. (2021) 14:e241712. doi: 10.1136/bcr-2021-241712 34183311 PMC8240584

[125] Salehi Kahyesh R Alghasi A Haddadi S Sharhani A . Intestinal parasites infection in children with cancer in Ahvaz, Southwest Iran. Interdiscip Perspect Infect Dis. (2020) 2020:8839740. doi: 10.1155/2020/8839740 33424965 PMC7775128

[126] Aguirre Fernandez MF Escobedo Belloc MA Moncada Flores KP . Atypical multisystemic manifestations of Ascaris infection in a patient with Burkitt lymphoma: a fatal diagnostic challenge. Cureus. (2026) 18:e101847. doi: 10.7759/cureus.101847 41717206 PMC12915696

[127] Ghosh S Roy K Rajalingam R Martin S Pal C . Cytokines in the generation and function of regulatory T cell subsets in leishmaniasis. Cytokine. (2021) 147:155266. doi: 10.1016/j.cyto.2020.155266 32888774

[128] Rajković M Deksne G Živković L Leonova E Spremo-Potparević B Sjakste N . DNA damage induced by parasitic infections in humans and animals. Comp Immunol Microbiol Infect Dis. (2025) 119:102337. doi: 10.1016/j.cimid.2025.102337 40220655

[129] Hatta T . Tick saliva molecules as potential immunomodulatory therapeutics. Parasitol Int. (2025) 112:103224. doi: 10.1016/j.parint.2025.103224 41423023

[130] Damane BP Mulaudzi TV Kader SS Naidoo P Savkovic SD Dlamini Z . Unraveling the complex interconnection between specific inflammatory signaling pathways and mechanisms involved in HIV-associated colorectal oncogenesis. Cancers (Basel). (2023) 15:748. doi: 10.3390/cancers15030748 36765706 PMC9913377

[131] Abou-El-Naga IF . Infection-driven autoimmune amplification versus molecule-specific immune modulation: the dual role of Toxocara canis in autoimmunity. Parasite Immunol. (2026) 48:e70085. doi: 10.1111/pim.70085 42138692

[132] Loredan DG Devlin JC Lacey KA Howard N Chen Z Zwack EE . Single-cell analysis of CX3CR1+ cells reveals a pathogenic role for BIRC5+ myeloid proliferating cells driven by Staphylococcus aureus leukotoxins. J Immunol. (2023) 211:836–43. doi: 10.4049/jimmunol.2300166 37466391 PMC10450158

[133] Pernaute-Lau L Adegnika AA Zhou Y Zinsou JF Gil JP Krishna S . Pharmacogene sequencing of a Gabonese population with severe Plasmodium falciparum malaria reveals multiple novel variants with putative relevance for antimalarial treatment. Antimicrob Agents Chemother. (2021) 65:e0027521. doi: 10.1128/AAC.00275-21 33875422 PMC8218688

[134] Fu F Yu Y Wang B Zhao X Wang N Yin J . Prostate and urinary microbiomes in prostate cancer development: focus on Cutibacterium acnes. Front Cell Infect Microbiol. (2025) 15:1562729. doi: 10.3389/fcimb.2025.1562729 40470262 PMC12133773

[135] Uram Ł Wróbel K Walczak M Szymaszek Ż Twardowska M Wołowiec S . Exploring the potential of lapatinib, fulvestrant, and paclitaxel conjugated with glycidylated PAMAM G4 dendrimers for cancer and parasite treatment. Molecules. (2023) 28:6334. doi: 10.3390/molecules28176334 37687164 PMC10489794

[136] Ding Y Zhang Z Ding C Xu S Xu Z . The use of cyclodextrin inclusion complexes to increase the solubility and pharmacokinetic profile of albendazole. Molecules. (2023) 28:7295. doi: 10.3390/molecules28217295 37959715 PMC10648351

[137] Kaewchim K Glab-Ampai K Mahasongkram K Chulanetra M Seesuay W Chaicumpa W . Engineered fully human single-chain monoclonal antibodies to PIM2 kinase. Molecules. (2021) 26:6436. doi: 10.3390/molecules26216436 34770845 PMC8588357

[138] Łanocha A Łanocha-Arendarczyk N Wilczyńska D Zdziarska B Kosik-Bogacka D . Protozoan intestinal parasitic infection in patients with hematological Malignancies. J Clin Med. (2022) 11:2847. doi: 10.3390/jcm11102847 35628973 PMC9146298

[139] Makipour H Haghighi A Halakou A Dayer D Bitaraf S Farhadi Kia A . Identifying zoonotic risks: molecular insights into Cryptosporidium and Enterocytozoon bieneusi in pediatric cancer patients in Ahvaz, 2024. Parasitol Res. (2025) 124:55. doi: 10.1007/s00436-025-08500-5 40407945 PMC12102135

[140] Pacifico T Tomassini L Biancone L Monteleone G Stolfi C Laudisi F . Repurposing rafoxanide: From parasite killer to cancer fighter. Biomedicines. (2025) 13:1686. doi: 10.3390/biomedicines13071686 40722758 PMC12292760

[141] Valenzuela-Salas LM Blanco-Salazar A Perrusquía-Hernández JD Nequiz-Avendaño M Mier-Maldonado PA Ruiz-Ruiz B . New protein-coated silver nanoparticles: Characterization, antitumor and amoebicidal activity, antiproliferative selectivity, genotoxicity, and biocompatibility evaluation. Pharmaceutics. (2021) 13:65. doi: 10.3390/pharmaceutics13010065 33430184 PMC7825588

[142] He W Baysal C Lobato Gómez M Huang X Alvarez D Zhu C . Contributions of the international plant science community to the fight against infectious diseases in humans-part 2: Affordable drugs in edible plants for endemic and re-emerging diseases. Plant Biotechnol J. (2021) 19:1921–36. doi: 10.1111/pbi.13658 34181810 PMC8486237

[143] Wondmagegn YM Setegn A Girmay G Abebe W Dessie N Ashagre A . Global prevalence of intestinal parasites in cancer patients: a systematic review and meta-analysis. BMC Infect Dis. (2025) 25:815. doi: 10.1186/s12879-025-11207-8 40596954 PMC12211656

[144] Arab-Mazar Z Kanani T Toulabi M Rouzbahani AK Fallahi S Behzadi A . Evaluation of strongyloidiasis prevalence in immunocompromised patients referred to hospitals: a case study of Iran’s capital. BMC Infect Dis. (2025) 25:381. doi: 10.1186/s12879-024-10431-y 40108519 PMC11921683

[145] Wagner M Koyasu S . Cancer in disguise: a parasite within. EMBO J. (2026) 45:1051–9. doi: 10.1038/s44318-025-00691-y 41530388 PMC12909802

[146] de Moura GA de Oliveira JR Rocha YM de Oliveira Freitas J Rodrigues JPV Ferreira VPG . Antitumor and antiparasitic activity of antimicrobial peptides derived from snake venom: a systematic review approach. Curr Med Chem. (2022) 29:5358–68. doi: 10.2174/0929867329666220507011719 35524668

[147] Cannon A Pajulas A Kaplan MH Zhang J . The dichotomy of interleukin-9 function in the tumor microenvironment. J Interferon Cytokine Res. (2023) 43:229–45. doi: 10.1089/jir.2023.0035 37319357 PMC10282829

[148] Silveira GO Coelho HS Amaral MS Verjovski-Almeida S . Long non-coding RNAs as possible therapeutic targets in protozoa, and in Schistosoma and other helminths. Parasitol Res. (2022) 121:1091–115. doi: 10.1007/s00436-021-07384-5 34859292

[149] Brühlmann F Perry C Griessen C Gunasekera K Reymond J-L Naguleswaran A . TurboID mapping reveals the exportome of secreted intrinsically disordered proteins in the transforming parasite Theileria annulata. mBio. (2024) 15:e0341223. doi: 10.1128/mbio.03412-23 38747635 PMC11237503

[150] Ahmetoglu D Zheng H Swart A Zhu H Li M . Multifaceted roles of guanylate-binding proteins in cancer. Int J Mol Sci. (2025) 26:5477. doi: 10.3390/ijms26125477 40564939 PMC12193362

[151] Xi Y Liu F Qiu B Li Y Xie X Guo J . Analysis of gut microbiota signature and microbe-disease progression associations in locally advanced non-small cell lung cancer patients treated with concurrent chemoradiotherapy. Front Cell Infect Microbiol. (2022) 12:892401. doi: 10.3389/fcimb.2022.892401 35719339 PMC9200620

[152] Mao QY Wang XQ Lin F Yu MW Fan HT Zheng Q . Scorpiones, scolopendra and gekko inhibit lung cancer growth and metastasis by ameliorating hypoxic tumor microenvironment via PI3K/AKT/mTOR/HIF-1α signaling pathway. Chin J Integr Med. (2024) 30:799–808. doi: 10.1007/s11655-024-3803-8 38850481

[153] Saki J Eskandari E Feghhi M . Study of toxoplasmosis and toxocariasis in patients suffering from ophthalmic disorders using serological and molecular methods. Int Ophthalmol. (2020) 40:2151–7. doi: 10.1007/s10792-020-01393-6 32424529 PMC7481152

